# Hurdles and signposts on the road to virtual control groups—A case study illustrating the influence of anesthesia protocols on electrolyte levels in rats

**DOI:** 10.3389/fphar.2023.1142534

**Published:** 2023-04-20

**Authors:** A. Gurjanov, A. Kreuchwig, T. Steger-Hartmann, L. A. I. Vaas

**Affiliations:** ^1^ Bayer AG, Pharmaceuticals, Investigational Toxicology, Berlin, Germany; ^2^ Bayer AG, Pharmaceuticals, Research and Pre-Clinical Statistics Group, Berlin, Germany

**Keywords:** historical control data, virtual control groups, systemic toxicity study, replacement, clinical chemistry, 3R

## Abstract

**Introduction:** Virtual Control Groups (VCGs) represent the concept of using historical control data from legacy animal studies to replace concurrent control group (CCG) animals. Based on the data curation and sharing activities of the Innovative Medicine Initiatives project eTRANSAFE (enhancing TRANSlational SAFEty Assessment through Integrative Knowledge Management) the ViCoG working group was established with the objectives of i) collecting suitable historical control data sets from preclinical toxicity studies, ii) evaluating statistical methodologies for building adequate and regulatory acceptable VCGs from historical control data, and iii) sharing those control-group data across multiple pharmaceutical companies. During the qualification process of VCGs a particular focus was put on the identification of hidden confounders in the data sets, which might impair the adequate matching of VCGs with the CCG.

**Methods:** During our analyses we identified such a hidden confounder, namely, the choice of the anesthetic procedure used in animal experiments before blood withdrawal. Anesthesia using CO_2_ may elevate the levels of some electrolytes such as calcium in blood, while the use of isoflurane is known to lower these values. Identification of such hidden confounders is particularly important if the underlying experimental information (e.g., on the anesthetic procedure) is not routinely recorded in the standard raw data files, such as SEND (Standard for Exchange of Non-clinical Data). We therefore analyzed how the replacement of CCGs with VCGs would affect the reproducibility of treatment-related findings regarding electrolyte values (potassium, calcium, sodium, and phosphate). The analyses were performed using a legacy rat systemic toxicity study consisting of a control and three treatment groups conducted according to pertinent OECD guidelines. In the report of this study treatment-related hypercalcemia was reported. The rats in this study were anesthetized with isoflurane.

**Results:** Replacing the CCGs with VCGs derived from studies comprising both anesthetics resulted in a shift of control electrolyte parameters. Instead of the originally reported hypercalcemia the use of VCG led to fallacious conclusions of no observed effect or hypocalcemia.

**Discussion:** Our study highlights the importance of a rigorous statistical analysis including the detection and elimination of hidden confounders prior to the implementation of the VCG concept.

## 1 Introduction

*In vivo* toxicity studies continue to play a central role in regulatory toxicology. The design of these studies is well harmonized: first, groups of animals are exposed to a test substance in different doses. This is followed by measurements, analyses and microscopic assessments of selected parameters (e.g., body weight, organ weights, electrolytes, protein levels) in the blood, serum, urine, tissues and organs (OECD, 2008; EMA, 2010; EMA, 2013; OECD, 2018a). To subsequently determine whether observed effects are treatment-related, the endpoints of the test substance-treated animals (i.e., treatment groups or dose groups) are compared to those of the control group. Statistical tests (such as Dunnett’s test (Dunnett, 1955)) are performed to determine the statistical significance of a deviation from the control data (Hamada, 2018). All measured data are then stored in the archives of the test facility, allowing the reuse of control group animal data for a historical control data (HCD) collection (Kluxen et al., 2021). So far, HCDs are mainly used as a reference base in toxicity studies (OECD, 2018b). Expert toxicologists use HCD to determine whether the measured endpoints in ongoing studies are within the range of HCDs. This helps in the assessment of biological relevance, i.e., if a certain measured value of dose group animals is outside the limits of the HCD values, this may indicate a biologically relevant effect (Kluxen et al., 2021). In most test facilities the HCD selection is only based on a fixed retrospective time interval but without any further statistical quality control.

The bioassays of regulatory toxicology are highly standardized, compliant to the guidelines of good laboratory practice (GLP) and conducted according to regulations of the US Food and Drug Administration (FDA), the European Medicines Agency (EMA), the Organization for Economic Cooperation and Development (OECD) and the International Council for Harmonization of Technical Requirements for Pharmaceuticals for Human Use (ICH). Given the highly controlled environment of the studies, it can be expected that the variations of physiological parameters between HCD and a concurrent control group (CCG) are limited and detectable in statistical analysis. Based on these considerations, the idea of the Virtual Control Groups (VCGs) was introduced some years ago (Steger-Hartmann et al., 2020). This concept aims to reduce the number of concurrent control group animals by using VCGs—which are generated from HCD—and thus to contribute to the 3R concept of (Russel and Burch, 1959).

The ViCoG (Virtual Control Group) was established as part of the Innovative Medicine Initiatives project eTRANSAFE (Pognan et al., 2021) with the aim of collecting, analyzing, and sharing HCD across multiple pharmaceutical companies and to start a qualification process for the VCGs. The collected HCD consist of animal data from regulatory toxicity studies conducted according to internationally highly standardized research practices (Bode, 2020) and their collection is often mandatory or strongly recommended (OECD, 2008; OECD, 2018a; OECD, 2018b; ICH, 2020). To use HCD for creating VCGs in the future, a common database meeting the following essential requirements has been set up: i) the collected data should have a harmonized format and ii) metadata must be recorded, such as information on the study design, animal suppliers, food type, and analytical methods.

Similar terminology and diagnostic criteria should be used when pooling data (Greim et al., 2003). Historically, a lack of harmonization has been a major obstacle to almost all data-collection efforts in Life Sciences (Kolker et al., 2012). But with the introduction of SEND (Standard for Exchange of Non-clinical Data) in 2002 (Wood and Lou, 2011) the harmonization of data from systemic toxicity studies has been greatly facilitated. SEND provides a set of harmonized terminologies and a framework for storing data of studies with various designs. Since 2016, SEND has been the mandatory format for submitting toxicity data to the FDA (CDISC, 2022), making it a well-suited framework for a database with a uniform architecture. With these SEND guidelines at hand, the members of the ViCoG can provide a large amount of historical control data in a harmonious format, which has been collected over the last decade. Beyond data harmonization, it is critical to obtain a thorough understanding of the characteristics and variability of the collected data itself before starting to replace CCGs with VCGs. Even minor changes in individual parameters, resulting from genetic drift or changes in methodological or analytical procedures, may induce significant differences between VCGs and concurrent controls impacting the outcome of statistical analyses and consequently have effects on the identification of treatment-related findings. In case of unknown or undocumented differences of design parameters influencing thus both the dependent and the independent variable, a classical confounder is present. To avoid misleading impact of those lurking variables, adequately matching of HCD for generation of VCGs is key. The importance has recently been shown for clinical pathology parameters (Wright et al., 2023) where the error rate in the recognition of treatment-related findings increased while loosening the selection criteria for HCD. In certain study types, such as carcinogenicity bioassays or the rat bone marrow micronucleus assays, requirements for the selection process of HCD are defined (Greim et al., 2003; Keenan et al., 2009; Igl et al., 2019).

OECD and ICH regulatory guidelines are less stringent when it comes to using HCD for comparison purposes but emphasize that data must be “collected from the same laboratory, species, strain and under similar conditions” (OECD, 2008; OECD, 2018b; OECD, 2018a), ideally also from “recent time” (ICH, 2020).

To summarize the requirements from literature and regulatory authorities, for *in vivo* toxicity studies discussed here, the following parameters are considered to be essential and therefore need to be controlled when comparing concurrent controls and HCD:

• sex,
• strain,
• supplier,
• age,
• housing conditions,
• route of administration,
• diet,
• tissue collection and processing procedures,
• treatment vehicle

and due to potential changes in analytical methods, one should also consider staying within a timeframe between 2 and 7 years.

These requirements provide a good starting point for the VCG approach in toxicity studies but given the huge number of measurements plus the complexity of employed bioassays, potential confounders remain highly likely. Constant and careful exploration shall be part of any activity creating robust and reliable VCGs.

Interestingly, the concept of using external controls instead of concurrent controls exists for clinical trials since years (Pocock, 1976) and has led to various applications to reduce the time- and resource-intensive recruitment of patients without risking the loss of statistical power or declining quality of the results. External controls are of particular interest for rare (orphan) diseases or for those where recruiting control subjects would be cumbersome or unethical (Pocock, 1976; Strayhorn, 2021). In the clinical setting, various methods have been introduced to derive external control groups from historical data that match subjects in a treatment arm of a clinical trial (Lim et al., 2018). Propensity score methods (Rosenbaum and Rubin, 1985), Bayesian methods (Lim et al., 2018; Zhan et al., 2022), or a mixture of both (Sawamoto et al., 2022) are used to construct external controls. Clinical trials with external controls generated by propensity scores have even successfully resulted in a drug approval (Gökbuget et al., 2016).

However, the methods for creating the VCGs from clinical studies cannot be directly transferred to non-clinical conditions. Clinical studies and preclinical animal experiments differ too much in terms of design and the homogeneity of their subjects. Control groups in clinical trials can show significantly higher variance in key characteristics such as age, body mass index, comorbidities, and especially genetic variance. In comparison, preclinical *in vivo* toxicity studies are usually designed as randomized case-control studies (e.g., control group and low, medium, and high dose group) and use treatment-naïve animals of similar, well-defined age, weight and low genetic variation. This is achieved by breeding under highly standardized conditions, strict inclusion criteria, and sourcing from the same supplier (White and Cham, 1998).

In the non-clinical settings, several methods for generating virtual control groups were proposed and include Bayesian approaches to either replace concurrent controls with inclusion of historical data (Kramer and Font, 2017; Wright et al., 2023)—which is the goal of the VCG approach—or to increase the statistical power of studies (Bonapersona et al., 2021). In regulatory toxicology the studies are conducted according to standardized guidelines (FDA, 2000; OECD, 2008; EMA, 2010), which provide general recommendations for group sizes for each study type. In short-term repeated-dose toxicity studies in rodents, a sample size of 10 animals per sex per group is normally recommended (FDA, 2000). Although statistical power is an important component for the design of meaningful assays (Charan and Kantharia, 2013), studies for preclinical safety assessment are carried out with those standardized designs and pre-specified group sizes. Considerations of individual statistical power or sample-size estimations play a minor role in preclinical safety assessment. This article therefore focuses on creation of virtual control groups with the goal of reducing the size of concurrent control group animals while maintaining the given design of the studies.

Aside from Bayesian approaches, simulation-based approaches that artificially generate control group values from aggregated historical data have also been proposed (Hothorn et al., 2019; Steger-Hartmann et al., 2020). However, the resulting VCG values would be purely synthetic and potential, but so far unknown, correlations between various endpoints might be difficult to reproduce. Therefore, a simple and straightforward resampling method (Steger-Hartmann et al., 2020), in which VCG data are randomly drawn directly from HCD build a meaningful first step here. The method is directly applicable and does not require complex mathematical and statistical background knowledge. In addition, the data shall come from historical studies conducted under tightly regulated GLP conditions and each value is directly traceable to the individual animal. Further, the outcomes of the resampling method are easy to interpret and allow for an in-depth analysis of the underlying data.

Before sampling the control group data, the HCD itself should be pre-filtered according to the key factors listed above to ensure concordance between concurrent controls and VCGs. It remains to determine how to validate the performance of the VCGs concept in general. Since regulatory toxicology works with strictly standardized studies, it is considered appropriate that VCG performance can initially be quantified by how well VCGs can reproduce the results of these studies. Thus, the VCGs need to reproduce results of a given historical study with respect to identifying treatment-related findings. In regulatory toxicology, significance tests are commonly performed to assess whether an observed difference is statistically significant or not (Hamada, 2018). When significance is detected, expert toxicologists determine whether the finding indicates a treatment-related effect by consulting HCD and observing interactions between various endpoints measured in the bioassay. HCD can be also used to calculate an effect size supporting the toxicologists in making this decision (Schmidt et al., 2016; Kluxen et al., 2021). However, because effect sizes were not calculated in the study reports reviewed in this article, we do not consider them here either and rather focus on VCGs’ ability to reproduce originally reported statistical significances of the studies.

In this article, a historical study of systemic toxicity in rats (legacy study) serves as a test case to evaluate and illustrate the performance of virtual control groups generated by a straightforward resampling method. A treatment-related increase in serum calcium was reported and VCGs should be able to reproduce this finding. Calcium belongs to the group of electrolytes where changes found in animal studies after administration of a drug candidate can help identify toxicities potentially leading to adverse events during clinical trials. Calcium is, apart from its role in bone formation, essential for the proper functioning of muscles, nerves, and the heart. Calcium imbalances can lead to severe effects such as bone pain, hypertension, seizures, tetany, paresthesia, laryngospasm, and cardiac conduction abnormalities (Schenck et al., 2006; Tinawi, 2021). The range considered normal is relatively small due to the strict physiological regulation of electrolytes. Therefore, small decreases or increases in serum concentrations are likely to result in significant differences between control and treatment groups.

We present VCGs generated by a resampling approach and validate their performance on the parameter calcium. The statistical significance of the selected legacy study serves as a reference for the performance and the aim is to reproduce the statistical results of this study, after replacing the CCGs with VCGs. During data quality assessment of the HCD, it was found that calcium, potassium, sodium, and phosphate values of control animals showed time-dependent changes that proved to be of critical importance in terms of proper data selection for VCGs. The presence of a confounder in the electrolyte values distorts their variability. This article demonstrates the consequences of an unreflective application of VCGs. Creating VCGs based on insufficiently prefiltered HCD results in a poor ability to reproduce statistical results which might in turn lead to erroneous toxicological decisions. We then describe strategies to counter the impact of such hidden confounders—and thus improving the performance of VCGs—by using adequate statistical control mechanisms. We developed a procedure for selecting data to be used for the generation of VCGs in toxicity tests and recommendations for an in-depth analysis that reveals previously unknown or unrecognized confounding variables.

Such a procedure would follow a stepwise approach: first, HCD needs to be selected to match common parameters such as study year, sex, strain, route of administration, treatment vehicle, supplier, age, and initial body weight. Afterwards, the quality of the resulting data must be assessed. This article shows that visualizing study data over time may reveal various phenomena in the data which offers a good starting point for identifying abnormalities, such as atypical shifts in values or unexplained increases or decreases in these values over time. Assuming that an atypical shift is detected in the data, an in-depth analysis is recommended to identify the root cause and underlying confounder in the data. In the event that the confounder cannot be identified, access to the original study reports and expert knowledge are critical for interpreting and tracing the confounder variables.

## 2 Methods

### 2.1 Data selection

All data were collected from previous animal studies performed by Bayer, Wuppertal, Germany. For these historical studies, the animals were kept and treated in accordance with the German Animal Welfare Act and approved by the competent state authorities. All data from animal studies were recorded in SEND format (Standard for Exchange of Non-Clinical Data) (CDISC, 2022). The data processing, statistical evaluations and visualizations were carried out with the software R, version 4.1.0. The R code used along with the data can be extracted from GitHub https://github.com/bayer-group/VCG-resampling.git. A detailed description of the origin of the data and the software used for data analysis can be found in the Supplementary Material S1, chapter 1.1 and 1.2. For the construction of the VCG database, control data sets for the different toxicological endpoints were extracted, including metadata describing the design of the study. The HCD were filtered with the aim of obtaining the largest possible amount of animal data while minimizing the potential impact of genetic variability. 28-day repeated dose toxicity studies are the dominant type of *in vivo* studies in regulatory toxicology (Baldrick, 2008; EMA, 2013). Therefore, data were selected from both 28-day studies and studies longer than 28 days in duration (in this case, only measured endpoints measured between study days 1 and 35 were extracted). Additional filter steps for studies for the VCG collection were selected based on the following criteria:

• Study initiation between 2011 and 2021.
• Usage of Wistar HAN rats.
• Age of rats between 6 and 9 weeks (at the beginning of the respective study).
• Animals obtained from the supplier Charles River, Germany, or Harlan, Netherlands.
• The route of administration was “oral gavage”.
• A mixture of Ethanol, Kolliphor®HS15, and water served as treatment vehicle.
• The initial body weight was between 100 g and 250 g.
• All endpoints were measured in Bayer’s laboratory in Wuppertal, Germany.
• Only male rats were used.

**FIGURE 1:**
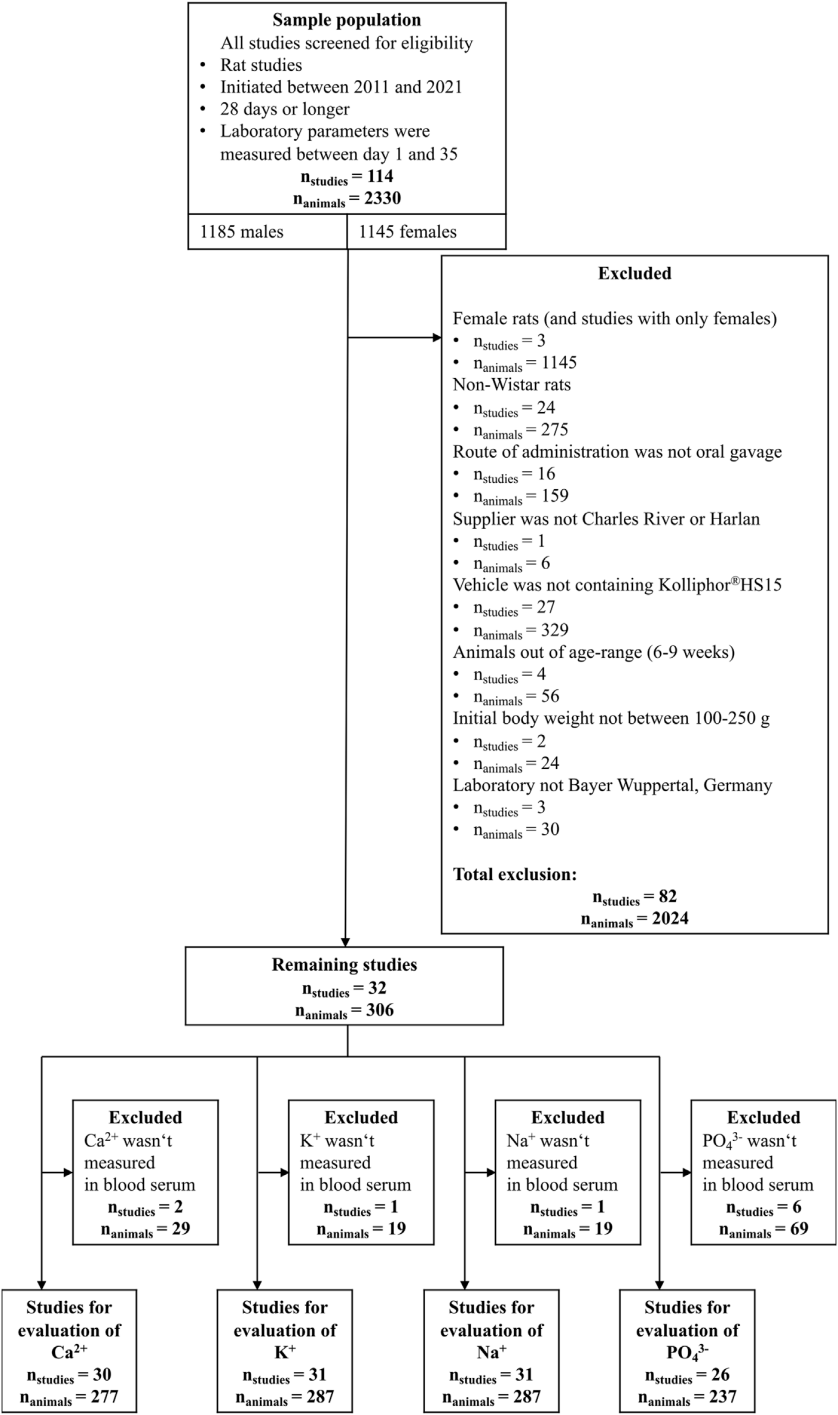
Data selection flow diagram.

From a total of 114 rat studies of the Bayer VCG data set, the data selection process reduced the set to 30 studies for calcium, 31 for potassium and sodium, and 26 studies for inorganic phosphate. The data selection process is summarized in Figure 1.

### 2.2 VCG performance assessment

To understand how replacing concurrent controls with VCGs influences the outcome of a study with respect to the so-called “treatment-relatedness” (Wright et al., 2023), a study with a reported treatment-related change in calcium in male rats was selected. This study is denoted as “legacy study” in this article. In the performance assessment, the aim was to test whether the statistical results of the legacy study were reproducible after replacing the concurrent control group (CCG) with VCGs while preserving the study design parameters of the legacy study. In other words, animals from the concurrent control group (CCG) of this legacy study were replaced by the same number of VCGs constructed by different selection criteria. Afterwards, the effect on the outcome was analyzed focusing on whether changes between control groups and dose groups were statistically significant (= evidence for a treatment-related effect) or not (= no evidence for a treatment-related effect). In addition to the calcium value, two other parameters were examined to gain an understanding of whether significant findings for correlated parameters can also be reproduced with the VCGs. Apart from the statistically significantly increased values for calcium, the legacy study also showed a significantly increased value in the highest dose group for the parameter inorganic phosphate in the blood serum. This parameter is strongly correlated with the calcium parameter, and it is of interest to test whether the VCGs can also reproduce this significance. Additionally, body weight on day 28 was taken into account. This parameter should not show strong correlations to the electrolyte levels in the rat and should not be affected by the used anesthetic.

**FIGURE 2:**
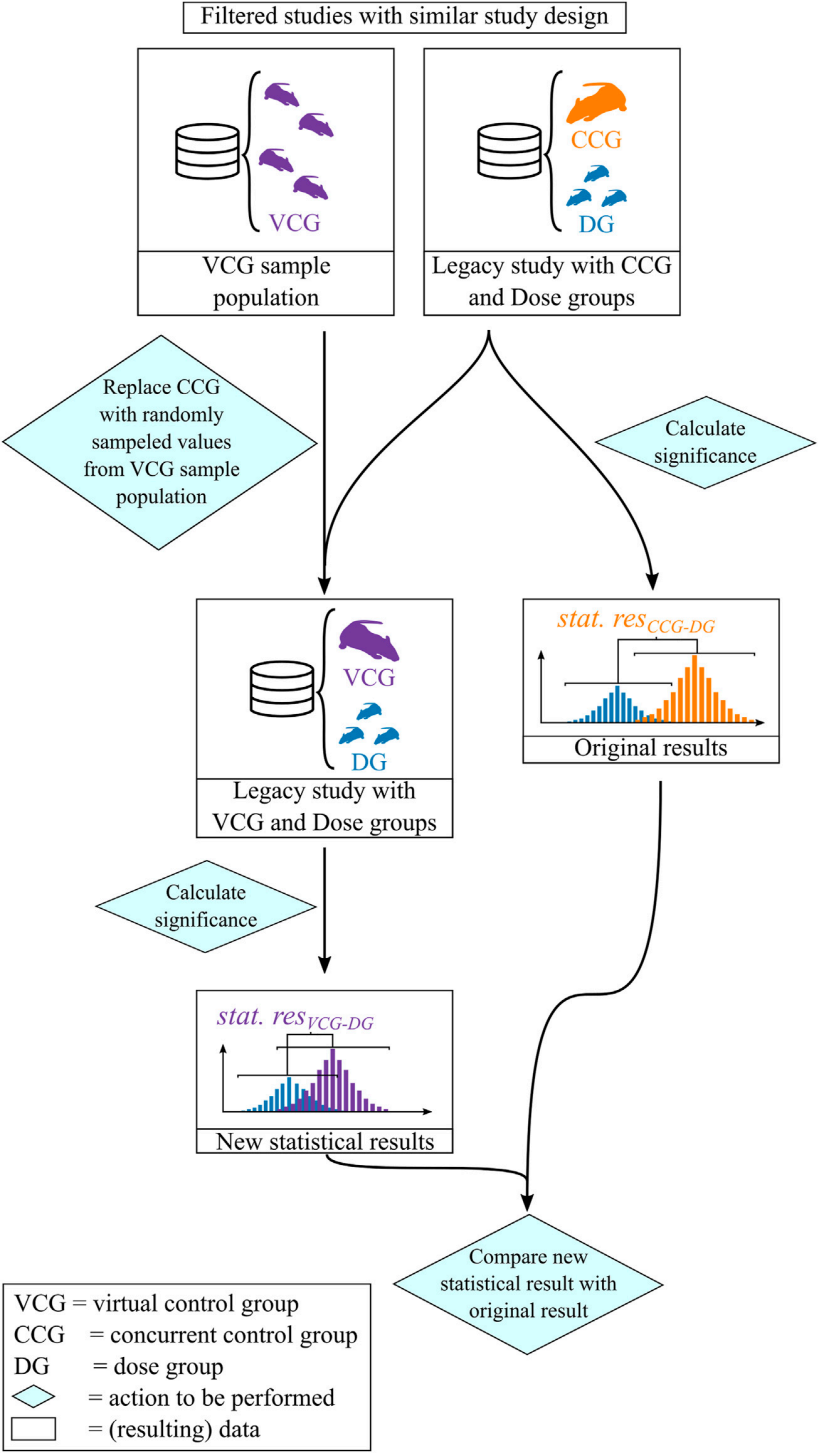
Workflow of assessing the performance of VCGs.

The resampling was performed in the following procedure which is illustrated in Figure 2:

1) The individual values for calcium in blood serum of the rats from the legacy study were retrieved from the database.
2) A Dunnett’s test was performed identical to the procedure used to analyze the original data with concurrent controls. The Dunnett’s test output was classified into two categories: if the resulting *p*-value of the Dunnett’s test was smaller than or equal to 0.05, the result was classified as “significant”, otherwise as “not significant”.
3) A sample population was created with respect to predefined filtering criteria.
4) *n* values were picked by random sampling without replacement from the sample population set where *n* is the number of removed CCG animals. The removed CCG-animal values were then replaced by these drawn VCG values.
5) The Dunnett’s test was calculated, now using the VCG instead of the CCG as the reference group.
6) The result of the Dunnett’s test was compared with the original outcome of the legacy study. If the VCG result was consistent with the CCG one, this sample was classified as “consistent”, otherwise as “inconsistent”. Inconsistent results were further classified into following categories:

• When the statistical outcome of the VCG leads to a significant result while the original distance was not significant, the result was classified as “inconsistently significant”.

• When the statistical outcome of the VCG leads to a non-significant result while the original distance was significant, the result was classified as “inconsistently non-significant”.

• When the statistical outcome of the VCG leads to a significant result and the original result does so too, but the direction of the distance is inverted (e.g., a significant decrease is observed while there was a significant increase in the original data), the result was classified as “inverted significant”.

7) The resampling was repeated 500 times and the percentage of consistent results per dose group was summarized. This percentage is termed here as the “reproducibility [%]” and is used as the parameter to validate the performance of the VCGs.

To examine the correlations of the VCGs with other parameters, the VCGs animals were selected based on their unique animal subject ID. This means that at each iteration, a certain number of animals were randomly sampled and the endpoints for all parameters of interest (i.e., calcium, phosphate, and body weight) were extracted from each selected animal.

### 2.3 Performance-improvement methods

#### 2.3.1 Search for confounders

The selected dataset was statistically analyzed for each parameter to gain an understanding of variance and distribution of the data over time. Electrolyte parameters were plotted as histograms and boxplots relative to the year the studies began. To gain further insight into how the underlying parameters (i.e., confounders) might affect electrolyte levels, the plots have been colored in relation to these confounders. Because procedural data for the identified confounder “anesthetic” was not captured in the SEND dataset, information was manually extracted from the selected study reports. In addition to the plots, a Welch-adapted *t*-test was performed to statistically describe the effects on the two groups separated by the confounder (the normality of the distribution with unequal variances was confirmed visually by the respective histograms). The VCG data was then filtered further: the VCG sample population was divided into animals anesthetized with isoflurane and animals anesthetized with CO_2_. After controlling for the confounder (i.e., using animals from only one of the anesthetic subgroups), the performance of the VCGs was re-evaluated by the same procedure as mentioned above.

#### 2.3.2 Keeping sentinel animals

Instead of replacing all CCG animals, the performance of the VCGs was additionally evaluated after only a fraction of the CCG animals were replaced. The CCG animals to remain (i.e., the sentinel animals) were selected using the initial body weight values of the CCGs, i.e., the body weight of the animals before the start of the study. The animals were selected with the aim of obtaining the original mean values and standard deviation values of the body weight distribution. Of *n* sentinel animals, the *n*/2 heaviest and the *n*/2 lightest animals remained in the control group, while the remaining animals were replaced with VCG data. In addition, when *n* was an odd number, an animal was selected from the middle of the initial body weight distribution, e.g., if it has been decided to include five out of ten animals, animal 1, 2, 5, 9 and 10 (sorted by body weight) were selected. Three different sub-scenarios were performed: i) all CCG animals were replaced by VCGs, ii) two animals were retained as sentinel animals, iii) half of the concurrent control animals remained in the group. To use all available information when recruiting VCGs, the measured calcium values of the sentinel animals were used as an additional filter narrowing down the VCG sample population: VCG data were filtered within the mean ± 2⋅SD range of sentinel animals’ calcium values. The performance of the resulting VCGs was then evaluated using the same design as described above and shown in Figure 2.

## 3 Results

**FIGURE 3:**
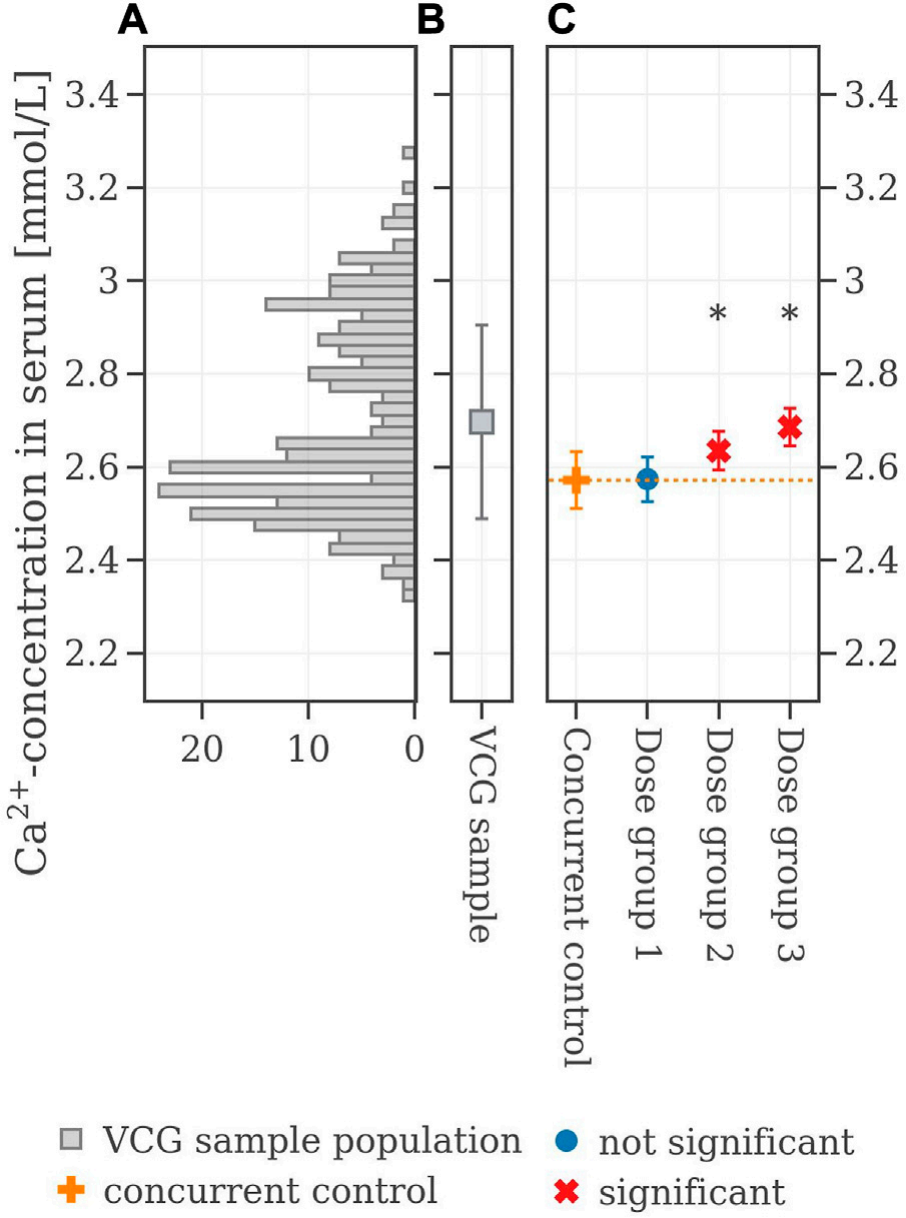
**(A)** Histogram of the calcium values of the VCG sample population. **(B)** Boxplot of the VCG sample population. **(C)** Extracted original results of serum calcium concentration from the legacy study. If the mean difference between a dose group and the concurrent control exceeded the Dunnett-critical distance, the group was marked with an asterisk.

This section is divided into the following parts: first, the statistical results of the original study with the concurrent control group are shown in Table 1 along with the distribution of the VCG sample population in Figure 3. The methods and the resulting performance of the VCGs are further separated in six scenarios: first, the performance of the VCGs (i.e., the reproducibility [%]) of the “agnostic scenario” is shown. Afterwards, the results for the approaches to improve the performance of the VCGs by two methods are presented: the “search for confounders” method where data was removed from the VCG sample population which was affected by a confounder; the “keeping sentinel animals” method where instead of completely replacing the CCG animal data either 80% or 50% of the animal data was replaced respectively; and finally, a combination of both methods where data affected by the confounder was removed and only 80% or 50% of all CCG animals were replaced. The results of all scenarios are summarized in Table 2. The ranges of the VCG data are illustrated in the Supplemetary Material S2, Supplemetary Figure S5.

**TABLE 1:** Selected legacy study with the mean calcium levels in blood serum in each sex, the standard deviation, and the population.

Dose group	Mean Ca^2+^ values in serum [mmol/L]	Population
Concurrent control	2.57 ± 0.06	10
Dose group 1	2.57 ± 0.05	10
Dose group 2	**2.64** ± 0.04*	10
Dose group 3	**2.69** ± 0.04*	10

*Significant difference with Dunnett *p*-value of <0.05.

Statistically significant differences between the respective dose groups and the control are highlighted in bold and marked with an asterisk.

**TABLE 2:** Resampling results of the legacy study on the parameter calcium after replacing the concurrent control group with virtual control groups (VCG) sampled from the respective subgroups. The sampling was performed 500 times and the percentage of consistent statistical results are given for each sex and each dose group (DG).

Mean value of the CCG [mmol/L]	Scenario	Mean value of the VCG sample population [mmol/L]	Sub-scenario	DG 1 consistency	DG 2 consistency	DG 3 consistency
2.57 ± 0.06	1: Confounder is unknown	2.69 ± 0.20	1a: Replace all CCG animals	**52% consistently non-significant**	**2% consistently significant**	**5% consistently significant**
					83% inconsistently non-significant	92% inconsistently non-significant
					15% inverted significant	3% inverted significant
				48% inconsistently significant		
			1b: Keep 2 sentinel animals	**90% consistently non-significant**	**100% consistently non-significant**	**100% consistently non-significant**
				10% inconsistently significant		
			1c: Replace half of the CCG animals	**100% consistently non-significant**	**100% consistently significant**	**100% consistently significant**
	2: Confounder is known	2 54 ± 0.08	2a: Replace all CCG animals	**74% consistently non-significant**	**97% consistently significant**	**100% consistently significant**
				26% inconsistently significant	3% inconsistently non-significant	
			2b: Keep 2 sentinel animals	**87% consistently non-significant**	**100% consistently significant**	**100% consistently significant**
				13% inconsistently significant		
			2c: Replace half of the CCG animals	**100% consistently non-significant**	**100% consistently significant**	**100% consistently significant**

The bold text represents the reproducibility percentage, i.e., the ability of the VCGs to reproduce the original statistical results of the legacy study and is thus a measure of performance of the VCGs.

### 3.1 Original statistical results of the legacy study

The legacy study consists of a concurrent control group (CCG) and three dose groups, denoted as Dose group 1, Dose group 2, and Dose group 3. All groups consisted of 10 male rats. The mean and standard deviation values as well as the population of the serum calcium values are shown in Table 1 and are illustrated in Figure 3. Statistical analysis employing Dunnett’s tests leads to no significant difference between CCG and Dose group 1 but revealed significant differences between the CCG and Dose groups 2 and 3 respectively. The corresponding historical control data for serum calcium values are shown as a histogram in Figure 3A: a bimodal distribution is present with two peaks at 2.87 mmol/L and 2.55 mmol/L. This renders the mean value of the VCG sample population to be at 2.68 ± 0.19 mmol/L (Figure 3B), i.e., considerably higher in mean value and standard deviation compared to the concurrent control with 2.57 ± 0.06 mmol/L (orange cross in Figure 3C).

### 3.2 VCG performance: The agnostic scenario

**FIGURE 4:**
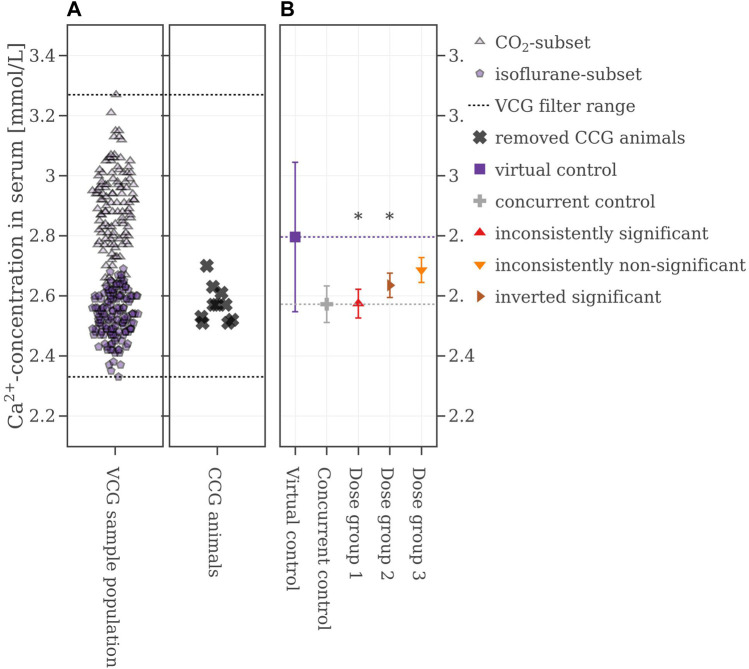
**(A)** Selection range of virtual control values and concurrent control values which are removed. **(B)** Mean values of the legacy study and the virtual control, colored with respect to whether the original statistical results were reproduced or not. If the mean difference between a dose group and the virtual control exceeded the Dunnett-critical distance, the group was marked with an asterisk.

In the first scenario, all control animals (*n =*10) were replaced and the VCG sample population was not further filtered ignoring both the bimodal distribution and the fact, that assays lacking controls would be invalid (Figure 4A). The consistencies for this approach were generally poor, with 52% for Dose group 1, 2% for Dose group 2, and 5% for Dose group 3 (see Table 2 for more details). One example iteration is shown in Figure 4B below: the mean value of the VCG (2.79 mmol/L) is considerably higher than the one of the CCG (2.57 mmol/L). Furthermore, while the mean values increased in comparison to the CCG with rising substance-level, here, the difference of mean values between the VCG and the dose groups is diminishing with increasing dose. Also, the standard deviation of the VCG (0.22 mmol/L) is considerably larger for the CCG (0.06 mmol/L) and the dose groups (0.05 mmol/L for Dose group 1, and 0.04 mmol/L for Dose groups 2 and 3 each). The mean values of the sampled VCGs from all iterations with respect to the reproducibility of the original results can be found in the Supplementary Material S2, Supplementary Figure S6.

### 3.3 Improvement of the performance: Search for confounders

**FIGURE 5:**
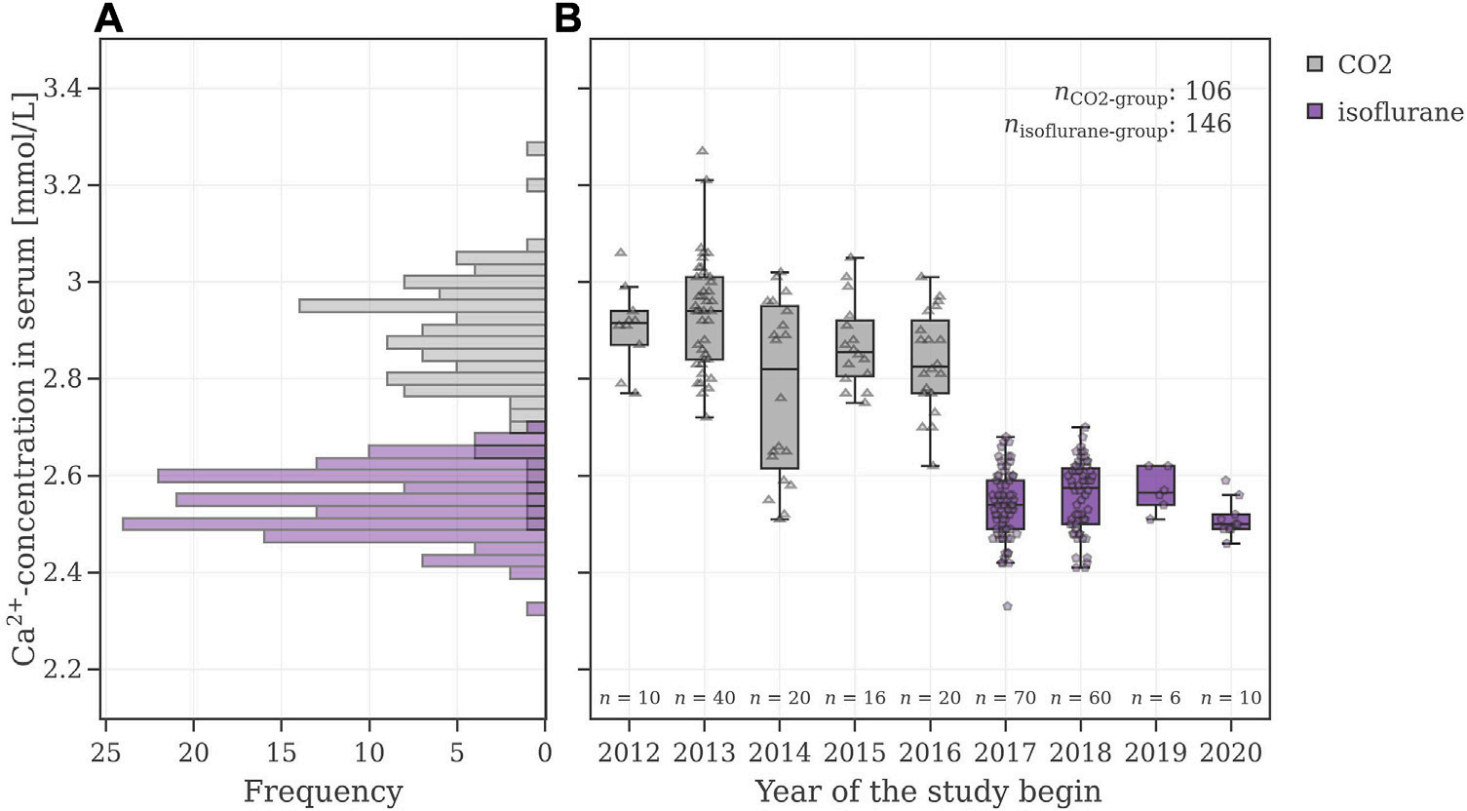
**(A)** Calcium value distributions of male Wistar-rats **(B)** Box plots of these calcium levels with respect to the study year. The CO_2_-group is colored grey, and the isoflurane-group is colored violet.

In order to understand the reason for the poor performance of the VCGs in the agnostic scenario, the very first step was to gain a deeper understanding of the underlying data and the factor(s) causing a bimodal distribution in the electrolytes. For a general overview of the control data pool, the electrolyte values were illustrated as histograms and as box plots with respect to the year when the study was initiated. Figure 5 shows the values for the electrolyte calcium. The electrolyte values for sodium, potassium, and phosphate can be found in the Supplementary Material S3, Supplementary Figures S12–S15. As mentioned before, a bimodal distribution in these electrolyte values was observed. The time-controlled graph revealed a sharp drop from 2016 to 2017. Before 2017, the mean values of calcium in serum were at (2.87 ± 0.14) mmol/L (95% CI [2.85, 2.90]) and dropped afterwards to (2.55 ± 0.07) mmol/L (95% CI [2.53, 2.56]).

An in-depth analysis revealed that before 2017 animals were anesthetized with a different procedure compared to animals in studies after 2017. As this information is not part of the SEND-data, the anesthetic procedure had to be extracted manually for each study. Implementing the information of the anesthetics revealed two normally distributed subsets shown in Figure 5: the distribution of the isoflurane subset (violet) has lower calcium levels and shows a smaller standard deviation (2.55 ± 0.07) mmol/L, while the distribution of the CO_2_ subset (grey) has higher calcium values and shows a higher/larger standard deviation (2.87 ± 0.14) mmol/L.

Finally, a Welch-adapted *t*-test was performed to assess the difference between these two groups. For calcium, the 95% CI for the difference in means resulted in [0.31, 0.37] with a *p*-value of <2.2 e−16.

**FIGURE 6:**
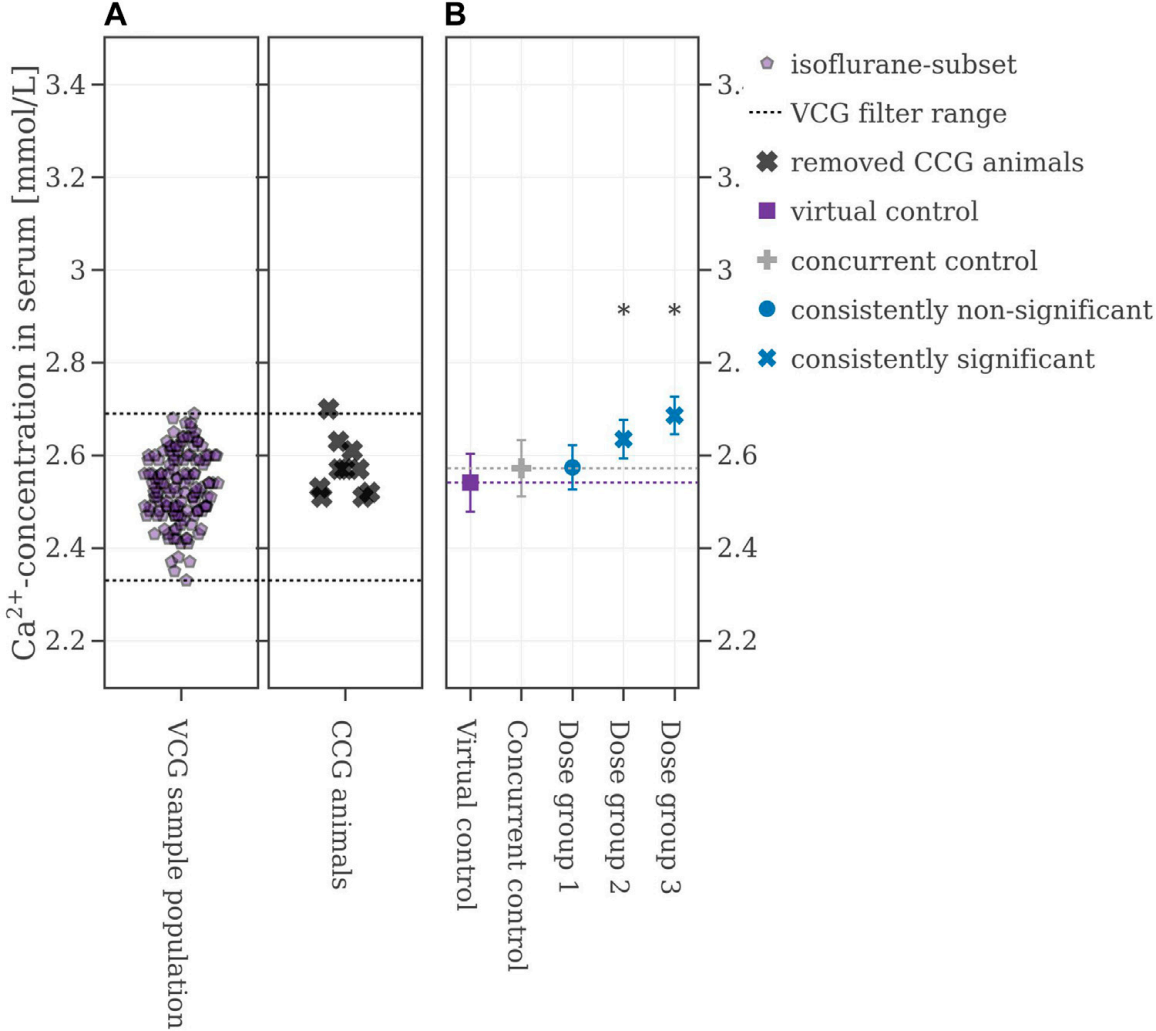
**(A)** Selection range of virtual control values along with concurrent control values which are removed. **(B)** Mean values of the legacy study and the virtual control, colored with respect to whether the original statistical results were reproduced or not. If the mean difference between a dose group and the virtual control exceeded the Dunnett-critical distance, the group was marked with an asterisk.

In the chosen legacy study, which was performed in 2018, the animals were anesthetized with isoflurane and subsequentially, the VCG sample population was selected accordingly by excluding data from animals anesthetized with CO_2_. The new range from which VCG animals were derived is shown in Figure 6A. Using this filter, the performance of the VCGs improved. Now, the consistencies were at 74% for Dose group 1, 97% for Dose group 2, and 100% for Dose group 3 (Table 2 for more details). Figure 6B shows the results from one of the 500 iterations. The CCG mean is (2.57 ± 0.06) mmol/L while the mean of the picked VCG is very close at (2.54 ± 0.08) mmol/L. The mean values of all iterations and with respect to the reproducibility of the original results can be found in the Supplementary Material S2, Supplementary Figure S9.

### 3.4 Improvement of the performance: Keeping sentinel animals

**FIGURE 7:**
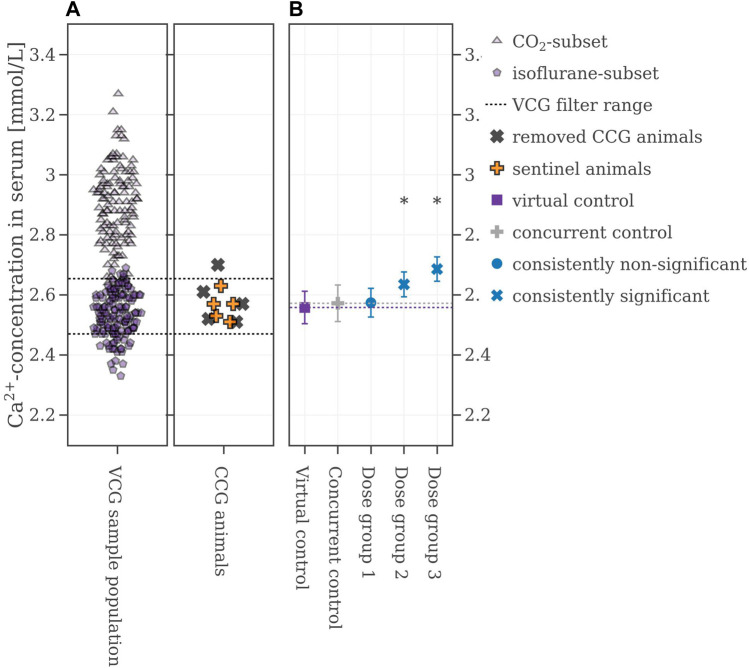
**(A)** Selection range of virtual control values along with concurrent control values which are removed and kept respectively. **(B)** Mean values of the legacy study and the virtual control, colored with respect to whether the original statistical results were reproduced or not. If the mean difference between a dose group and the virtual control exceeded the Dunnett-critical distance, the group was marked with an asterisk.

As a second option to improve the performance of the VCGs several concurrent animals of the legacy study were kept as so-called sentinel animals. After filtering for heaviest and lightest animals, their calcium values’ mean ± 2∙SD range was used to narrow down the VCG sample population. Two scenarios were examined here: keeping the values from two CCG animals and keeping the values of half of the CCG population (shown in Figure 7A). If two animals were kept and VCGs were only derived within the calculated range, the consistency of the VCGs was at 90% for Dose group 1, and 100% for Dose group 2 and 3 respectively. Keeping half of the CCG animals in the set improved the performance to 100% for all dose groups respectively (see Table 2 for more details). Figure 7B shows one example iteration of the performed 500 where half of the animals were kept as sentinel animals. The CCG mean value is at 2.57 ± 0.06 mmol/L while the mean value of the VCG is again very close to the CCG at 2.56 ± 0.06 mmol/L. The mean values of the VCGs from all iterations with respect to the reproducibility of the original results can be found in the Supplementary Material S2, Supplementary Figure S7, S8.

### 3.5 Improvement of the performance: Control confounder and keep sentinel animals

Combining both methods did not considerably improve the performance. Keeping two sentinel animals and controlling for the confounder had a similar performance as the one where only the confounder was controlled: 87% of all iterations led to reproducible results for Dose group 1, 100%, and 100% for Dose group 2 and 3. Keeping half of the CCG animals while controlling for the confounder led to a high consistency of 100% for all dose groups respectively (Table 2 for more details). The mean values of all sampled VCGs with respect to the reproducibility of the original results for all iterations can be found in the Supplementary Material S2, Supplementary Figures S10, S11.

### 3.6 The performance of the VCGs on the parameter inorganic phosphate and body weight

The same performance patterns could be observed for phosphate: in the “agnostic scenario”, a generally poor performance is recorded, with 11% for Dose group 1, 18% for Dose group 2, and 4% for Dose group 3. However, the performance was significantly increased by both improvement methods. Using only isoflurane data improved the performance considerably: the statistical results from all iterations were reproduced in 62% for Dose group 1, 80% for Dose group 2, and 83% for Dose group 3. Sentinel animals selected based on the calcium parameter also increased performance on the phosphate parameter. Keeping two sentinel animals in the control group revealed a reproducibility of 95% in Dose group 1, 99% in Dose group 2, and 66% in Dose group 3. With five sentinel animals retained, a reproducibility of 100% was found for Dose groups 1% and 2, and 82% for Dose group 3 was observed. Further details can be found in the Supplementary Material S4, Supplementary Table S1.

The parameter body weight on day 28 could generally be reproduced well. In the agnostic scenario, the statistical results were reproduced with 94% for Dose group 1% and 100% for Dose group 2 and 3. After removal of CO_2_ data from the VCG sample population, the reproducibility even decreased to 76% for Dose group 1, and 95% for Dose group 2. Dose group 3 remained at 100%. The presence of sentinel animals left the original performance unchanged. The statistical results for Dose group 1 could be reproduced in 95% of all iterations when two sentinel animals were kept; Dose groups 2 and 3 in 100% of all cases. Five sentinel animals resulted in a reproducibility of 100% in all dose groups. Further details on the performance of the VCGs towards the body weight parameter can be found in the Supplementary Material S4, Supplementary Table S2.

## 4 Discussion

In this article, we describe key requirements for statistical characterization of HCD prior to the use of historical data for the implementation of VCGs. Through time-control plots of electrolyte values we identified a sudden drop from 1 year to the other and were able to identify changes in the anesthetic procedure as cause of this drop. We subsequently analyzed how such a hidden confounder might influence the replacement of CCG with VCGs with regard to identification of treatment-related effects using a legacy study, in which treatment-related findings for calcium were reported. This was demonstrated by the performance of the virtual controls, which was assessed by their ability to reproduce the statistical significance of the increased calcium values from the legacy study. The performance of the VCGs is poor when using a insufficiently filtered data set and increased impressively after removing the confounding factor and any data affected by that confounder.

### 4.1 Assessment of VCG performance

The performance is a percentage resulting from the comparison of *p*-values obtained from the Dunnett’s test—once between the CCG and the dose groups of the legacy study and once between the VCGs and the dose groups of the same study. This procedure was repeated 500 times and at each iteration animals were drawn at random from the VCG sample population. Though the statistical significance is not the only decisive factor for toxicologists to speak of a treatment-related effect, it is nevertheless a cornerstone for further decision-making. It is therefore the first sensible step to test whether the statistical significance of the legacy study can be reproduced with the VCGs. The aim was to keep the design of this study unchanged, except for the CCG values which have been replaced by VCGs. The design of the legacy study itself was carried out in compliance with the guidelines for toxicity studies (FDA, 2000; OECD, 2008; EMA, 2010). The FDA guideline states that “for short-term toxicity studies of 30 days duration or less, experimental and control groups should have at least 10 rodents per sex per group” which the legacy study adhered to. To maintain the study design, any removed CCG value was replaced with exactly one value drawn from the VCG sample population, resulting in a constant number of *n* = 10 control animals. Although not considered in this article, increasing the number of control animals by introducing historical data might be an effective way to improve statistical power (Bonapersona et al., 2021). Scenarios with different control group sizes could be examined for potential changes in statistical power, and finally, for discussion of how increasing the power could be beneficial for decision-making in regulatory toxicology. However, a prerequisite would be strict and carefully chosen specifications for the selection of appropriate data together with expert knowledge input from both statisticians and regulatory toxicologists. Recommendation here is a selection of HCD from studies which are as similar as possible in design to the current study. A list of design parameters is proposed (Supplementary Material S5, Supplementary Table S3) and should be expanded in the future with ongoing research on definition of clear inclusion and exclusion criteria. The inclusion of VCGs from animals from different study designs might not necessarily increase statistical power or help toxicologists deciding whether observed statistical significances are treatment related. For example, an article on the curation and analysis of histopathological parameters in a large database showed that the proportions of pathological findings may change significantly with increasing sample size (Pinches et al., 2019). Although histopathological data are qualitative parameters, a similar conclusion could be drawn for quantitative parameters: a large HCD population could increase data variability and alter statistical results. There is currently no minimum number of *in vivo* toxicity studies recommended by the OECD to generate meaningful HCDs. However, for *in vitro* studies such as the mammalian micronucleus test, a minimum of ten studies is required (OECD, 2016). It might be worth to assess in future what the minimum number of studies would be for generation of meaningful VCGs. However, relying on HCD alone in a toxicological evaluation might be problematic since, compared to HCD, the concurrent data for bioassay interpretation are generally considered to be the most relevant as they ensure assay validity and help to identify infections in cages (Keenan et al., 2009; Kluxen et al., 2021).

**FIGURE 8:**
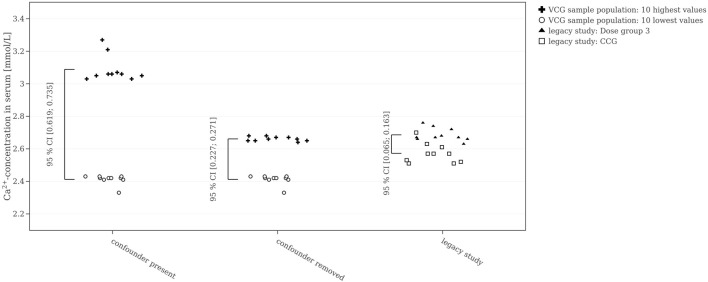
10 highest and 10 lowest calcium values of the Virtual Control Group (VCG) sample population (once with and once without the values affected by the confounder) compared to the Concurrent Control Group (CCG) and Dose group 3 of the legacy study. The 95% confidence interval for the difference in means is shown for each group.

A further suggestion is to check how representative HCD is to simulate concurrent controls. Here, we focused on reproducing the statistical significance of the chosen legacy study and monitor only one endpoint. Regulatory toxicology studies look for both target and off-target effects of a substance screening a comparably large number of endpoints without exerting an *a priori* defined endpoint hierarchy. When it comes to decision-making, an important aspect are relevance limits (Schmidt et al., 2016; Kluxen et al., 2021). To assess the size of potentially detectable effects in a given scenario with HCD, the confidence interval for the largest possible difference in the means of the HCD as the relevance limit is shown in Figure 8. Based on the ten largest and ten smallest calcium values of the HCD sample population, a 95% CI for the difference of means using a two-sample *t*-test is calculated. The legacy study presented here reports evidence of hypercalcemia determined by expert toxicologists. For reproduction of this treatment-related finding, the difference between the mean values of CCG and Dose group 3 would have to be greater than the one between the extreme values from HCD. In the legacy study, the 95% confidence interval for the difference of means between the CCG and Dose group 3 (i.e., the high dose group) obtained by a two-sample *t*-test was [0.065; 0.163] mmol/L. Calculating Cohen’s D between these two groups resulted in an effect size of 2.2. For the VCG sample population, the difference in means between the 10 highest calcium levels and the 10 lowest calcium levels resulted in a mean difference of [0.619; 0.735] mmol/L when the confounder was present, i.e., about six times higher compared to the difference between CCG and Dose group 3. Cohen’s D-calculation resulted in a large effect size of 11.0, also, five times higher than the effect observed in the legacy study. Removing all values affected by the confounder, selecting the 10 highest and 10 lowest calcium values, and recalculating the *t*-test reduced the difference in means to [0.227; 0.271] mmol/L, still two to three times greater than the difference between CCG and Dose group 3. However, the effect size was still large at 10.8.

Consequently, considering the differences in means or Cohen’s D as the limit of biological relevance for estimating a biological effect is not straightforwardly applicable. Communication between toxicology experts and regulatory authorities is still needed to decide on acceptable limits and selection criteria of parameters to determine a standardized effect size (EFSA Scientific Committee, 2011).

### 4.2 Identifying hidden confounders and retaining sentinel animals dramatically increases VCG performance

Replacing all concurrent control animals with virtual control data from an insufficiently pre-filtered sample population (i.e., the “agnostic scenario”) resulted in poor overall performance. In this scenario, a confounder was present that elevated the mean value and the variance of the VCG sample population. This strongly increased variance led to a non-significant statistical result in most cases and this means in turn that the significant differences in Dose groups 2 and 3 from the legacy study cannot be reproduced. To improve the performance of the VCGs compared to the “agnostic scenario”, two methods were presented: finding the parameter in the study design that influences the outcome of the study, and keeping a certain number of sentinel animals in the concurrent control group.

To find the confounder, the data of the VCG population was plotted against the study year and searched for atypical shifts. A marked drop in electrolyte levels was observed in the VCG sample population in 2017 (Figure 5), which ultimately compromised the performance of the VCGs. A confounder was present but could not be identified immediately. After excluding known stratification parameters—such as strain (Kacew, 1996), route of administration (Gad, 1994), sex, age (Wolford et al., 1987; McCutcheon and Marinelli, 2009), initial body weight (Wolford et al., 1987), vehicle (de Kort et al., 2020) and the laboratory carrying out the test (Igl et al., 2019)—other parameters were checked as possible reasons, such as a change in animal supplier and a potential change in the analytical method. However, none of these parameters provided an explanation for the observed decline in electrolytes in 2017. Discussing the data with the study director led to the identification of an additional parameter, which is not recorded in SEND, namely, the anesthetics used before blood collection. Since blood collection is a stressful procedure for animals, anesthesia with CO_2_ or isoflurane is required by animal welfare ordinance (Parasuraman et al., 2010). While CO_2_ is a cheap and easy to use gas that does not require resource intensive disposal, isoflurane is considered to be less stressful for the animals (Altholtz et al., 2006; Traslavina et al., 2010; Wong et al., 2013; Turner et al., 2020). But apart from that, it is also documented that CO_2_ as an anesthetic can artificially increase blood serum electrolyte levels, as high levels of CO_2_ cause blood acidosis (Langford, 2005; Traslavina et al., 2010). Isoflurane, on the other hand, is known to lower electrolyte levels (Hotchkiss et al., 1998; Deckardt et al., 2007). Manual extraction of this information from the original reports and subsequent addition to the database finally confirmed that the change from CO_2_ to isoflurane caused the observed changes in electrolytes. Afterwards, the common VCG database was enriched with this parameter in order to generate meaningful VCGs for the simulation of electrolyte parameters.

Aside from finding and controlling confounders, the performance of VCGs has been increased after keeping a certain number of sentinel animals in the CCG set. Concurrent control animals are generally essential to ensure the quality and technical validity of the bioassay (Kluxen et al., 2021). For example, in a study without control animals, a possible infection would go unnoticed and a resulting increase in hematological parameters could be incorrectly attributed to the administered test item (Nicklas et al., 1999; Steger-Hartmann et al., 2020). Already keeping two sentinel animals significantly improved the performance of the VCGs compared to the “agnostic scenario” and keeping half of the CCG animals as sentinel animals resulted in a reproducibility percentage of 100% in all dose groups (see Table 2).

The combination of both methods, i.e., controlling the stratification parameter and keeping sentinel animals, did not improve the performance of the VCGs with respect to the method of “keeping sentinel animals”. Sentinel animals attenuated the influence of the confounder in the presented study. This emphasizes their usefulness beyond ensuring the technical validity of a bioassay.

This article demonstrates that rigorous control of the data increases the performance of the VCGs. However, future VCG selection and matching should not be based on solely one parameter and should use several parameters instead. An long-term goal is to create VCGs from historical data that ideally are able cover all endpoints of a toxicological study well. In regulatory toxicology, around 75 quantitative clinical pathology parameters (and other qualitative ones, e.g., histopathology) are examined. Examination of each of these parameters for quality, as has been presented for the electrolyte values in this article and a comprehensive analysis of the parameter distribution at a particular time of measurement would make it possible to identify further confounders and thus continuously improve the quality of the HCD and generate more meaningful VCGs. Another factor to consider is the correlation or interdependence of several parameters. Calcium is known to correlate with phosphate, urea and potassium (Howard et al., 2011; Verzicco et al., 2020), and parathyroid hormone (PTH) (Lecoq et al., 2021), among others. If a parameter was found to differ significantly, it might also be of interest to check whether a correlating endpoint also shows a significant change in the same direction. If so, these findings could be flagged accordingly, which could ultimately help expert toxicologists and study directors differentiate true treatment-related effects from artifacts. In the legacy study, the above-mentioned correlation of calcium and inorganic phosphate (both are increased) were present. Both electrolytes are regulated by Vitamin D3, PTH, and calcitonin (Shrimanker and Bhattarai, 2016). The VCGs—selected to match the parameter calcium—show the same behavior for phosphate as for calcium: a poor performance when the confounder is present and an increase in performance after either leaving sentinel animals in the set or removing the data affected by the confounder (Supplementary Material S4, Supplementary Table S1). Thereby, in this case, we were able to reproduce the statistical results of the study well. In addition, VCGs were examined for a parameter that does not primarily correlate with blood serum electrolyte levels: the body weight, measured on day 28 at the end of the dosing period (Supplementary Material S4, Supplementary Table S2). No significant changes in body weight concentration were noted in the legacy study between the dose groups and the corresponding CCG. Unsurprisingly, the VCGs were able to reproduce these results well as they were selected leaving the original location parameters of the initial body weights of the rats unchanged. Neither removing the data affected by the confounder nor considering sentinel animals did affect the good performance of the VCGs.

## 5 Conclusion

Our study illustrates the importance of proper data analysis and selection and proposes strategies to generate virtual controls. The aim of this study was to generate VCGs to replace concurrent controls in future experiments, thereby contributing to the 3R concept. VCGs were generated by a resampling approach that proved to be easy to implement and straightforward, yielding results that were easy to interpret. In addition, each individual VCG value can be traced back to each individual animal in a historical study allowing for quality assurance. The performance of the VCGs was shown to be highly dependent on the quality of the underlying HCD and can be improved by using a small number of remaining concurrent control animals (i.e., sentinel animals). A well maintained and constantly improved database together with thorough statistical characterization of the data will be the main requirements for the implementation of VCG.

## Data Availability

The datasets presented in this study can be found in online repositories. The names of the repository/repositories and accession number(s) can be found below: GitHub: https://github.com/bayer-group/VCG-resampling.git.
