# Supplementary material for: Hurdles and signposts on the road to virtual control groups—A case study illustrating the influence of anesthesia protocols on electrolyte levels in rats
Source: Front Pharmacol. 2023 Apr 20;14:1142534. doi: 10.3389/fphar.2023.1142534 (PMC10159271; doi:10.3389/fphar.2023.1142534)
Supplement: Supplementary file 1 [file DataSheet4.docx]

Supplementary Material

# Results

In the supplementary Table S1 we show the VCG performance for the parameter inorganic phosphate, a parameter strongly correlated with calcium which is the main endpoint observed in this article. The legacy study which is used here as the reference reported a significantly increased phosphate value in Dose group 3 (i.e., the high dose group) while Dose group 1 and 2 did not show any significant differences. The reproducibility % shows the same behavior in this endpoint as observed in calcium (see Table 2 of the main body of the article). Namely, the performance of the “agnostic scenario” is very poor due to the presence of a confounder. Upon removing all data points affected by the confounder, the performance increased considerably. The presence of sentinel animals improved the performance even further. Note that the VCG was filtered to match the sentinel animals in the parameter calcium. Phosphate—being strongly correlated to calcium—profited from this additional filtering step as well.

Apart from phosphate, another parameter was observed where it wasn’t expected that the anesthetic procedure (being a confounder for electrolyte values measured in blood serum) would affect the values: the body weight measured on day 28 of the study (supplementary Table S2). This parameter indeed didn’t show any improvements in performance after removing all data from animals affected by the confounder. However, keeping five sentinel animals in the set of the concurrent control group improved the performance slightly.

# Supplementary Tables

Table S1: Resampling results of the legacy study on the parameter phosphate after replacing the concurrent control group with virtual control groups (VCG) sampled from the respective subgroups. The sampling was performed 500 times and the percentage of consistent statistical results are given for each sex and each dose group (DG).

| DG 3 consistency | **4 % consistently significant**  93 % inconsistently non-significant  3 % inverted significant | **66 % consistently significant**  34 % inconsistently non-significant | **82 % consistently significant**  18 % inconsistently non-significant | **83 % consistently significant**  17 % inconsistently non-significant | | **97 % consistently significant**  3 % inconsistently non-significant | **100 % consistently significant** |
| --- | --- | --- | --- | --- | --- | --- | --- |
| DG 2 consistency | **18% consistently non-significant**  82 % inconsistently significant | **99 % consistently non-significant**  1 % inconsistently significant | **100 % consistently non-significant** | **80 % consistently non-significant**  20 % inconsistently significant | | **100 % consistently non-significant** | **99 % consistently non-significant**  1 % inconsistently significant |
| DG 1 consistency | **11 % consistently non-significant**  89 % inconsistently significant | **95 % consistently non-significant**  5 % inconsistently significant | **100 % consistently non-significant** | **62 % consistently non-significant**  38 % inconsistently significant | | **99 % consistently non-significant**  1 % inconsistently significant | **100 % consistently non-significant** |
| Sub-scenario | 1a: Replace all CCG animals | 1b: Keep 2 sentinel animals | 1c: Replace half of the CCG animals | 2a: Replace all CCG animals | | 2b: Keep 2 sentinel animals | 2c: Replace half of the CCG animals |
| Mean value of the VCG sample population [mmol/L] | 2.30 ± 0.65 | | | | 1.94 ± 0.31 | | |
| Scenario | 1: Confounder is unknown | | | | 2: Confounder is known | | |
| Mean value of the CCG [mmol/L] | 1.52 ± 0 28 | | | | | | |

Table S2: Resampling results of the legacy study on the parameter body weight (on day 28) after replacing the concurrent control group with virtual control groups (VCG) sampled from the respective subgroups. The sampling was performed 500 times and the percentage of consistent statistical results are given for each sex and each dose group (DG).

| DG 3 consistency | **100 % consistently non-significant** | **100 % consistently non-significant** | **100 % consistently non-significant** | **100 % consistently non-significant** | | **100 % consistently non-significant** | **100 % consistently non-significant** |
| --- | --- | --- | --- | --- | --- | --- | --- |
| DG 2 consistency | **100 % consistently non-significant** | **100 % consistently non-significant** | **100 % consistently non-significant** | **95 % consistently non-significant**  5 % inconsistently significant | | **99 % consistently non-significant**  1 % inconsistently significant | **100 % consistently non-significant** |
| DG 1 consistency | **94 % consistently non-significant**  6 % inconsistently significant | **95 % consistently non-significant**  5 % inconsistently significant | **100 % consistently non-significant** | **76 % consistently non-significant**  24 % inconsistently significant | | **96 % consistently non-significant**  4 % inconsistently significant | **100 % consistently non-significant** |
| Sub-scenario | 1a: Replace all CCG animals | 1b: Keep 2 sentinel animals | 1c: Replace half of the CCG animals | 2a: Replace all CCG animals | | 2b: Keep 2 sentinel animals | 2c: Replace half of the CCG animals |
| Mean value of the VCG sample population [g] | 303 ± 23 | | | | 295 ± 21 | | |
| Scenario | 1: Confounder is unknown | | | | 2: Confounder is known | | |
| Mean value of the CCG [g] | 319 ± 24 | | | | | | |
