# Supplementary material for: Hurdles and signposts on the road to virtual control groups—A case study illustrating the influence of anesthesia protocols on electrolyte levels in rats
Source: Front Pharmacol. 2023 Apr 20;14:1142534. doi: 10.3389/fphar.2023.1142534 (PMC10159271; doi:10.3389/fphar.2023.1142534)
Supplement: Supplementary file 2 [file DataSheet3.docx]

Supplementary Material

# Results

## Electrolyte values with respect to the used anesthetic

This section illustrates the differences in the electrolyte values of control-group animals from studies with as a histogram and as box plots with respect to the study year. The graphs are separated by color to illustrate the different anesthetics used in the studies. In each electrolyte value, namely calcium (Figure S12), potassium (Figure S13), sodium (Figure S14), and inorganic phosphate (Figure S15) there is a bimodal distribution visible in the histogram as well as a drop in the box plots from 2016 to 2017, *i.e.*, the year when the anesthetic procedure was changed from CO_2_ to isoflurane.

# Supplementary Figures


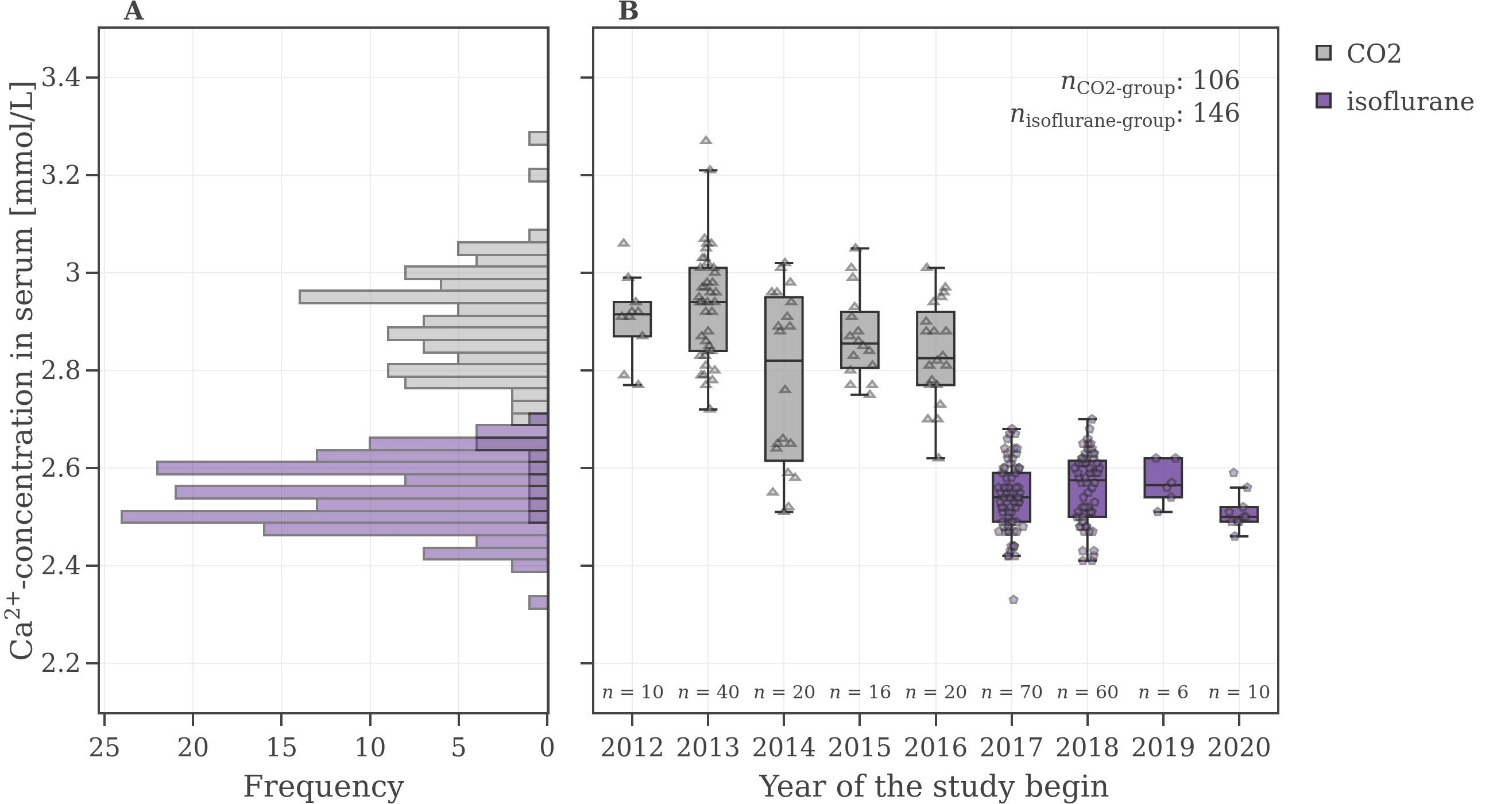


Figure S12: (A) Calcium value distributions of male Wistar-rats (B) Box plots of these calcium levels with respect to the study year. (C) Calcium values as box plots with respect to the anesthetic. The CO_2_-group is colored grey, and the isoflurane-group is colored violet.


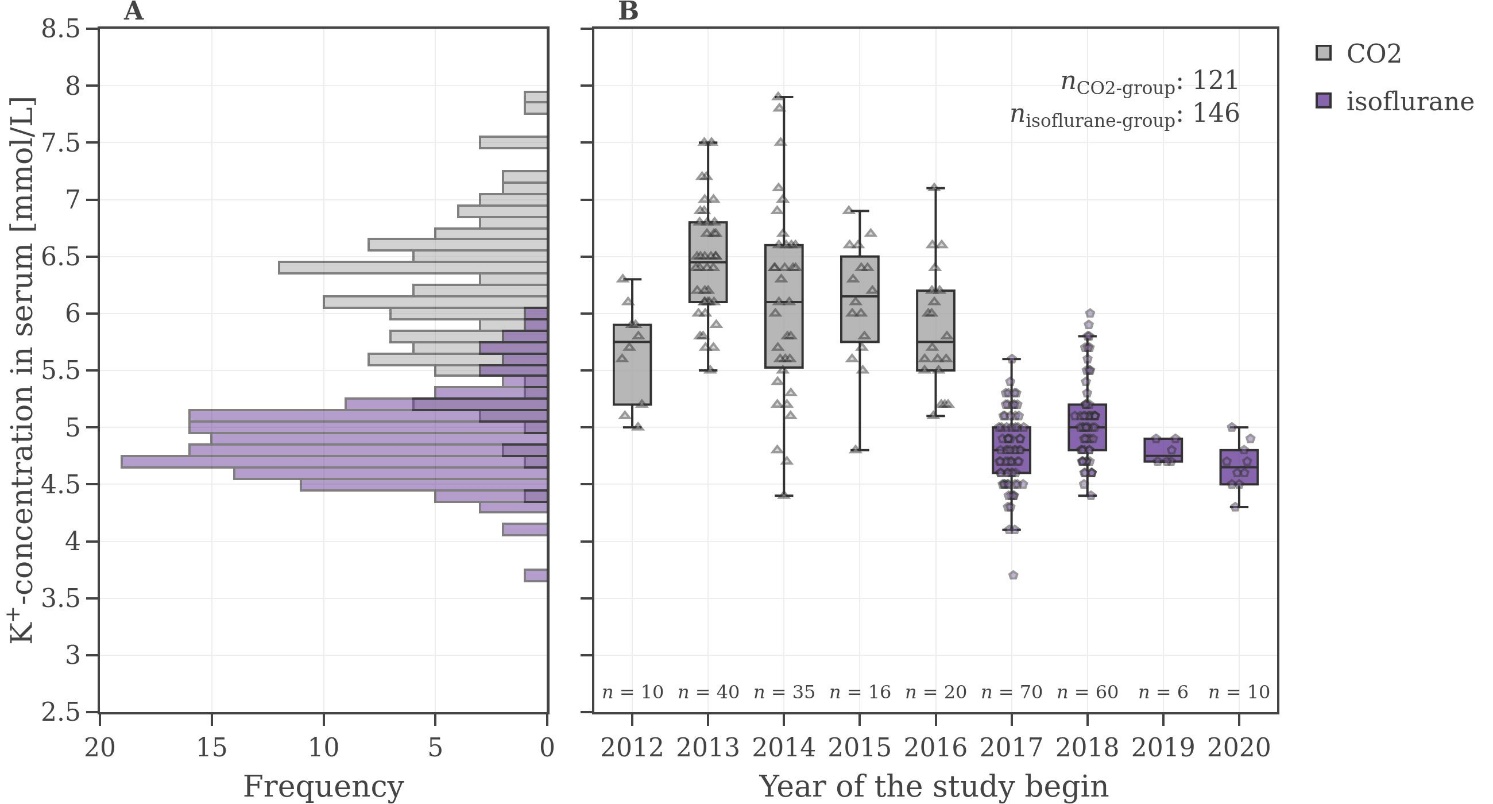


Figure S13: (A) Potassium value distributions of male Wistar-rats (B) Box plots of these potassium levels with respect to the study year. (C) Potassium values as box plots with respect to the anesthetic. The CO_2_-group is colored grey, and the isoflurane-group is colored violet.


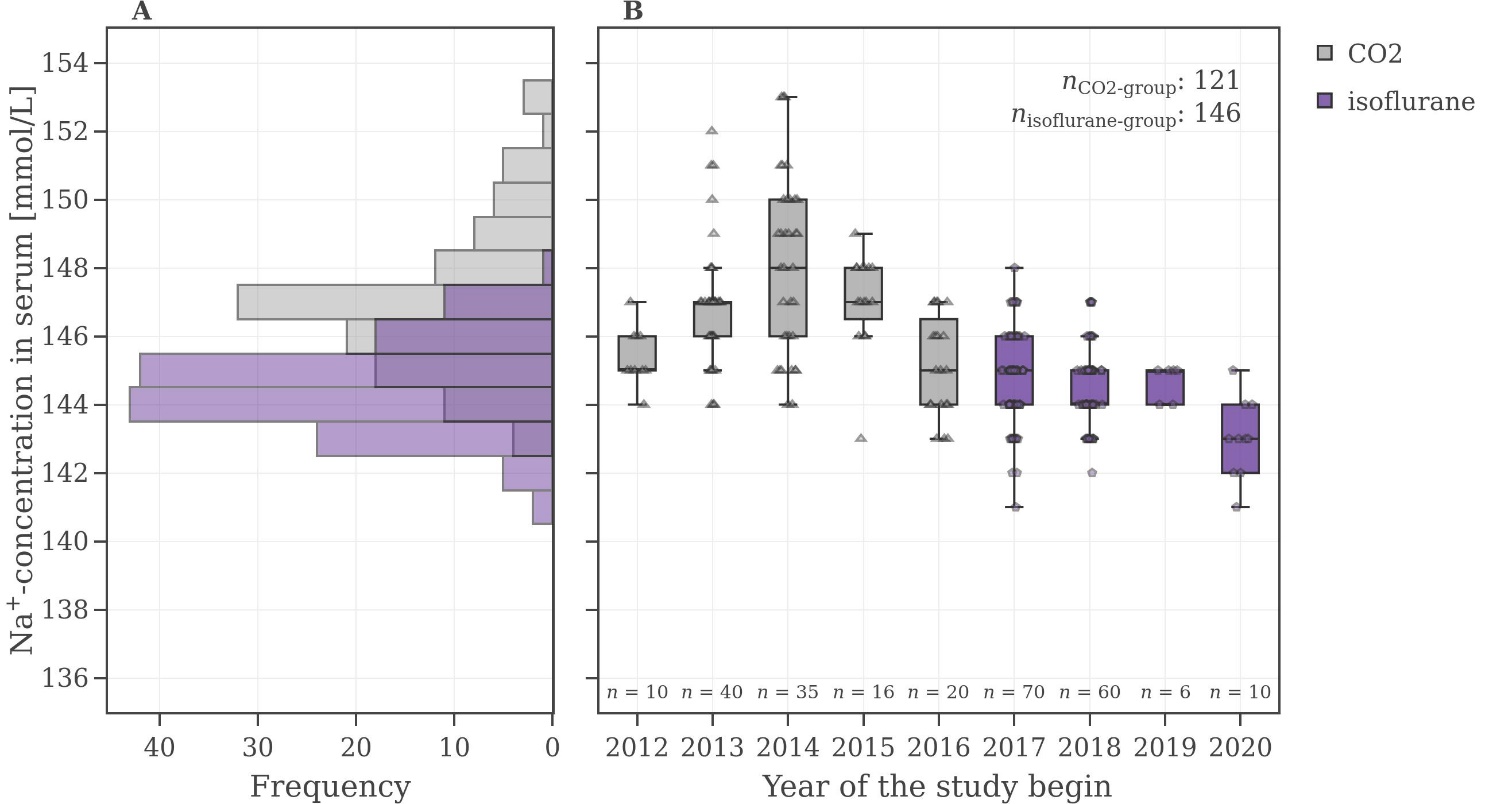


Figure S14: (A) Sodium value distributions of male Wistar-rats (B) Box plots of these sodium levels with respect to the study year. (C) Sodium values as box plots with respect to the anesthetic. The CO_2_-group is colored grey, and the isoflurane-group is colored violet.


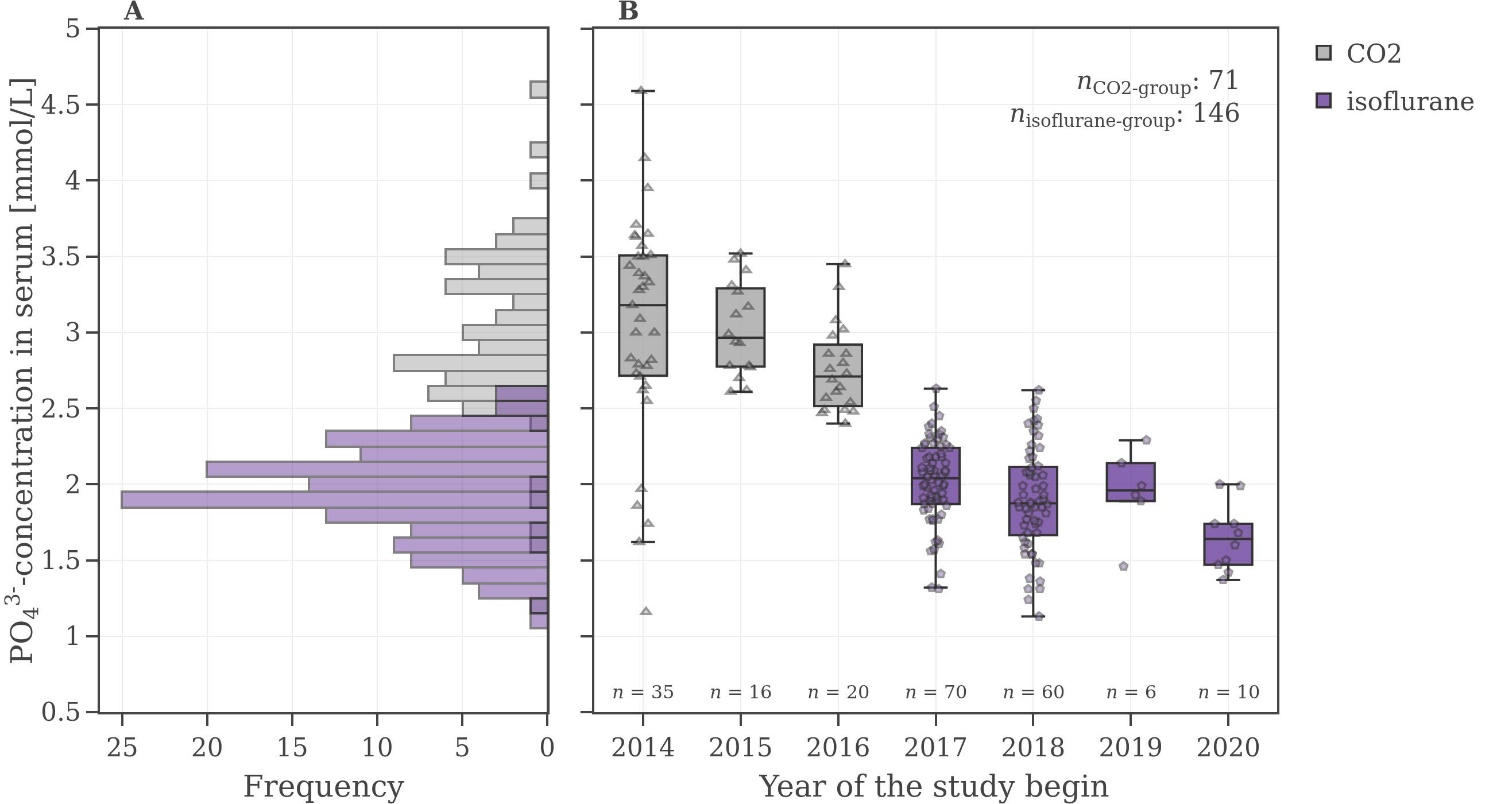


Figure S15: (A) Phosphate value distributions of male Wistar-rats (B) Box plots of these phosphate levels with respect to the study year. (C) Phosphate values as box plots with respect to the anesthetic. The CO_2_-group is colored grey, and the isoflurane-group is colored violet.
