# Supplementary material for: Hurdles and signposts on the road to virtual control groups—A case study illustrating the influence of anesthesia protocols on electrolyte levels in rats
Source: Front Pharmacol. 2023 Apr 20;14:1142534. doi: 10.3389/fphar.2023.1142534 (PMC10159271; doi:10.3389/fphar.2023.1142534)
Supplement: Supplementary file 3 [file DataSheet5.docx]

Supplementary Material

# List of study design parameters proposed to use as filters for VCG selection.

In the supplementary Table S3 we present a set of parameters we propose to control in order to derive meaningful virtual controls.

# Supplementary Tables

Table S3: List of study design parameters proposed to use as filters for VCG selection.

| **Parameter (SEND-name)** | **Selected value** | **Rationale** |
| --- | --- | --- |
| **Essential** | | |
| Species (SPECIES) | RAT | Animals differ strongly in their parameters. |
| Strain (STRAIN) | WISTAR HAN | Different strains, differ in various parameters.(de Kort et al., 2020) |
| Route of administration (ROUTE) | ORAL GAVAGE | Different routes of administration may alter the outcome of parameters (*e.g.,* skin findings after intravenous injections)(Gad, 1994). |
| Dosing duration (DOSDUR) | at least 28 d | Studies shorter than 4 weeks often are performed for exploratory purposes in a non-standardized setting where not all parameters of a GLP 4-week study are monitored. |
| Study day (BWDY/LBDY) | 1 - 35 | It was decided to observe only the study days 1 - 28, however 1 week was added in case that some time passed between finishing the dosing period and measuring respective parameters. |
| **Aspect to be considered** | | |
| Study start-year (STDSTDTC) | 2018 – 2022 | Genetic drift may introduce variation of parameters over time, which should be prevented. In addition, analytical methods may change over time (e.g., flame photometry vs. Ion-selective electrodes for determination of cations) |
| Initial age (AGE) | 6 weeks – 9 weeks | Aging affects many parameters rats. However, the age entries are rather vague. In addition, animals are usually ordered according to weight and not age. Thus, the body weight should be used as a surrogate for age.(Wolford et al., 1987; de Kort et al., 2020) |
| Body weight (BWORRES) (as a surrogate for age) | 100 g – 250 g | Controlling body weight prevents entering abnormally young/old animals in the set which might influence other parameters (e.g., ALT, ASP, GGT).  It is proposed to select a body weight distribution similar to the one reported for the study under investigation. |
| Supplier/Breeder (SPLRNAM) | CHARLES RIVER | There is weak evidence for a change in supplier which may influencing parameters (potassium, calcium). Further investigations needed. |
| Test facility location (TSTFLOC) | E.g., Bayer Pharmaceuticals, Wuppertal | The laboratory has a high impact on different measured endpoints and it is recommended by the guidelines to use historical data from only one laboratory.(OECD, 2008; Keenan et al., 2009)  While taking data from only one laboratory is preferable, certain endpoints might still be enriched by consulting data from different laboratories as well as long as GLP criteria are met. |
| **Useful** | | |
| Treatment vehicle (TRTV) | KolliphorHS15 | Different vehicles may have a different impact on the metabolism of the animals.(Stokes et al., 2013; de Kort et al., 2020) |
| Time from beginning of the substance treatment until sacrifice of animals (TRMSAC). | 1 - 35 | Unlike the dosing duration, this parameter takes the “recovery period” into account. During this time, control animals are not exposed to the treatment vehicle which can have an outcome on certain values. |

# References

de Kort, M., Weber, K., Wimmer, B., Wilutzky, K., Neuenhahn, P., Allingham, P., et al. (2020). Historical control data for hematology parameters obtained from toxicity studies performed on different Wistar rat strains: acceptable value ranges, definition of severity degrees, and vehicle effects. Toxicology Research and Application 4, 2397847320931484.

Chang, W. (2019). webshot: Take Screenshots of Web Pages. R package version 0.5.2. https://CRAN.R-project.org/package=webshot.

Dowle, M., and Srinivasan, A. (2021). "data.table: Extension of `data.frame`. R package version 1.14.0. https://CRAN.R-project.org/package=data.table".).

Gad, S.C. (1994). Routes in toxicology: An overview. Journal of the American College of Toxicology 13(1), 34-39.

Keenan, C., Elmore, S., Francke-Carroll, S., Kemp, R., Kerlin, R., Peddada, S., et al. (2009). Best practices for use of historical control data of proliferative rodent lesions. Toxicologic pathology 37(5), 679-693.

OECD (2008). Test No. 407: Repeated Dose 28-day Oral Toxicity Study in Rodents.

Sievert, C. (2018). "plotly for R. https://plotly-r.com".).

Signorell, A., and al., e.m. (2019). "DescTools: Tools for descriptive statistics. R package version 0.99.28. https://cran.r-project.org/package=DescTools".).

Stokes, A.H., Kemp, D.C., Faiola, B., Jordan, H.L., Merrill, C.L., Hailey, J.R., et al. (2013). Effects of Solutol (Kolliphor) and cremophor in polyethylene glycol 400 vehicle formulations in Sprague-Dawley rats and beagle dogs. International journal of toxicology 32(3), 189-197.

Wickham, H. (2017). "tidyverse: Easily Install and Load the 'Tidyverse'. R package version 1.2.1. https://CRAN.R-project.org/package=tidyverse".).

Wolford, S., Schroer, R., Gallo, P., Gohs, F., Brodeck, M., Falk, H., et al. (1987). Age-related changes in serum chemistry and hematology values in normal Sprague-Dawley rats. Fundamental and Applied Toxicology 8(1), 80-88.
