# Supplementary material for: Hurdles and signposts on the road to virtual control groups—A case study illustrating the influence of anesthesia protocols on electrolyte levels in rats
Source: Front Pharmacol. 2023 Apr 20;14:1142534. doi: 10.3389/fphar.2023.1142534 (PMC10159271; doi:10.3389/fphar.2023.1142534)
Supplement: Supplementary file 4 [file DataSheet2.docx]

Supplementary Material

# Results

## Legacy study graphical results

This section shows the overall results of all 500 iterations of all six scenarios used to test the VCG performance. All recruitment scenarios are shown in Figure S9. For the numerical results of the performances for each scenario please refer to Table 2 of the main body of this article. The graphs illustrate the mean values of the VCGs in each iteration and the outcoming result for each dose group. In the first scenario (1A), *i.e.*, the “agnostic scenario”, which is shown in Figure S10, a high variance of the mean values of the VCGs can be seen. As a result, virtual controls with a mean value too far away from the concurrent one tends to lead to results inconsistent to the one of the legacy study. The performance of the VCGs was improved by two approaches. One approach was to keep a number of values from the legacy study of satellite animals in the set. Keeping two satellite animals (Figure S11) reduced the variance of the VCG means and improved the performance considerably; keeping five animals (*i.e.*, half of the original control group population) (Figure S12) improved the performance even further. Another strategy was to control the confounding factor (*i.e.*, the anesthetic used) as shown in Figure S13 which too, improved the performance, though not as good as keeping sentinel animals. Combining both methods, *i.e.*, controlling the confounder and keeping two sentinel animals (Figure S14) or five sentinel animals (Figure S15) did not further improve the performance of the virtual controls.

# Supplementary Figures


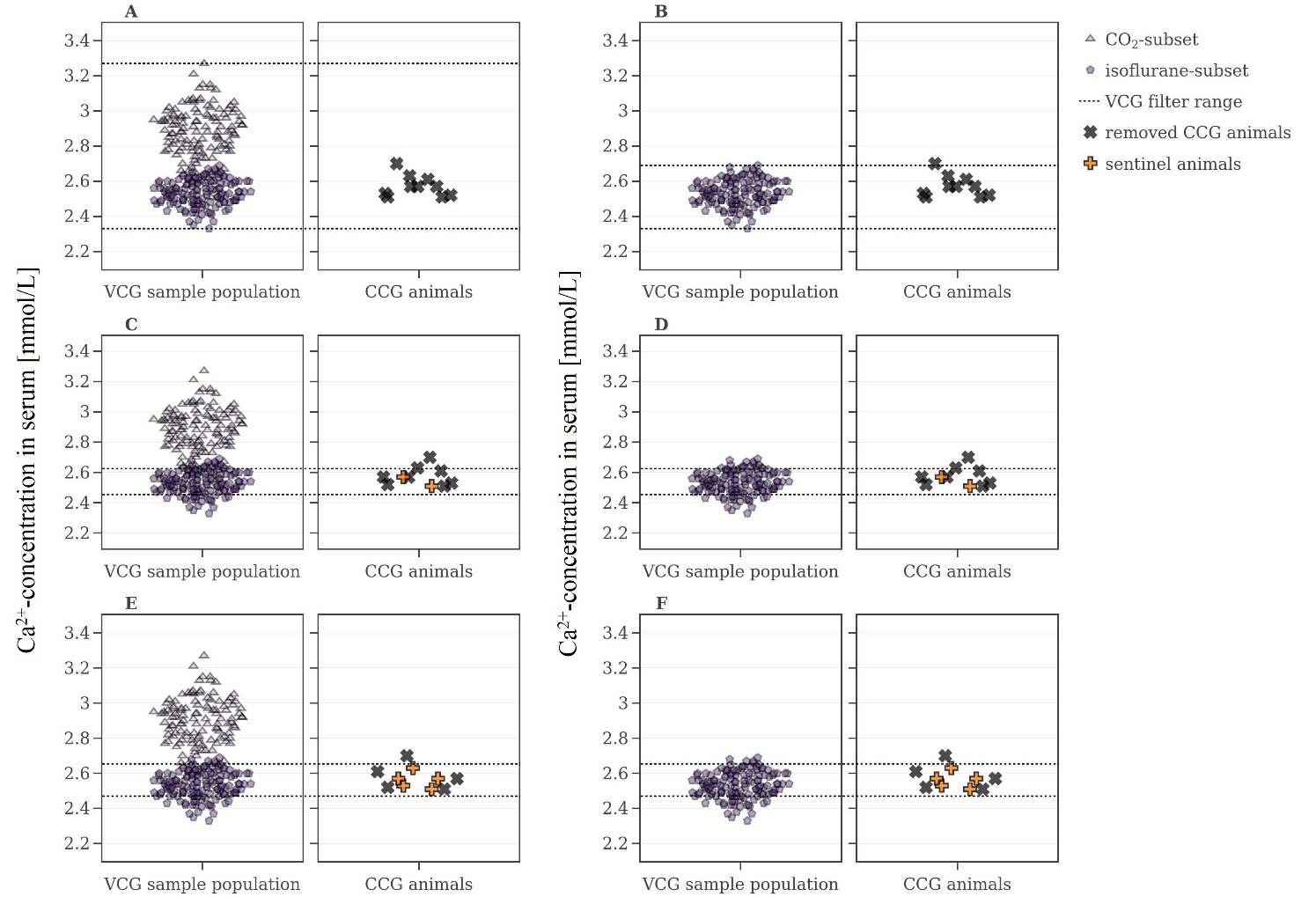


Figure S5: All scenarios of the VCG selection process. (A) The “agnostic-scenario”) No sentinel animals kept; confounder not controlled. (B) No sentinel animals kept; confounder controlled. (C) 2 sentinel animals kept; confounder not controlled. (D) 2 sentinel animals kept; confounder controlled. (E) Half of the CCG-animals kept; confounder not controlled. (F) Half of the CCG animals kept; confounder controlled.


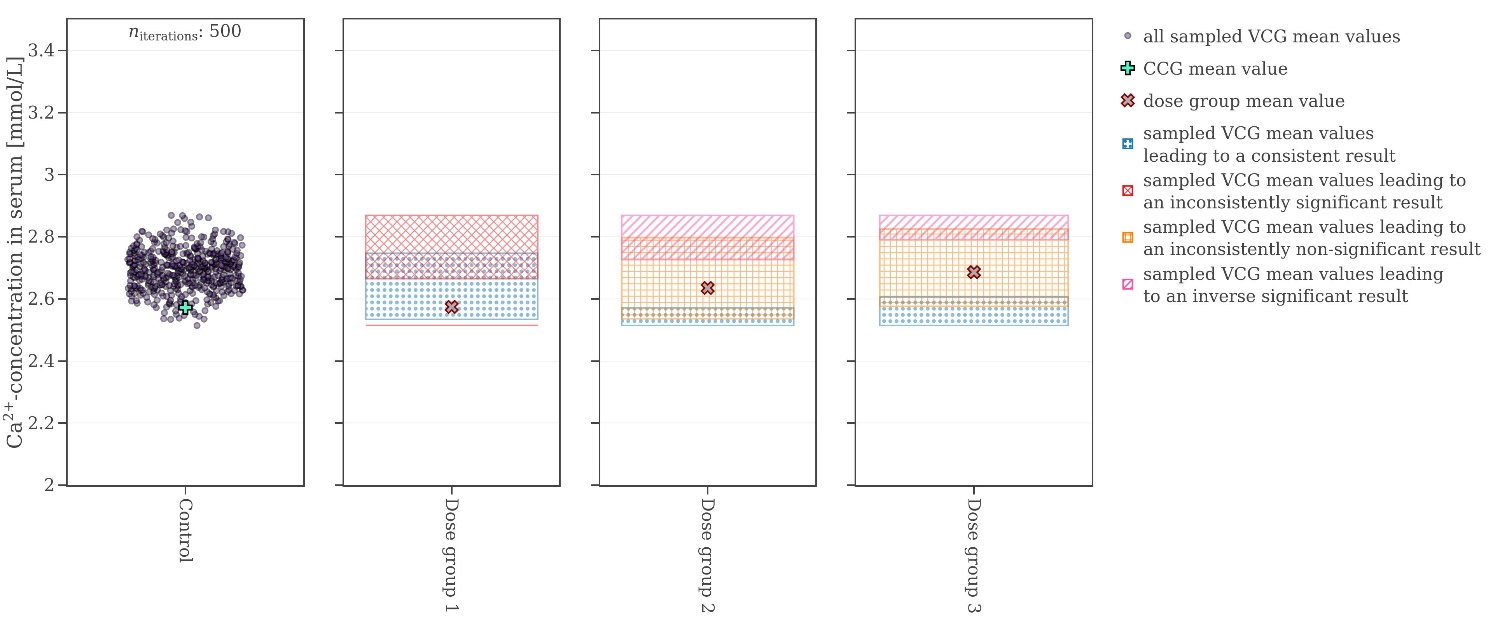


Figure S6: Scenario 1a: confounder not controlled; no sentinel animals kept. Resampling results of the legacy study in male rats in each dose group. Virtual control groups (VCGs) were generated using the calcium sample population of the respective subset The mean value of concurrent control group (CCG) (green cross) and the dose group (grey X) are shown for each dose group. Additionally, all mean values of the sampled VCGs of 500 iterations are shown as a scattered violet cloud in the control-group panel. On the dose groups, the areas for the VCG from each iteration are shown as “zones”. If a VCG led to a result consistent with the one using the CCG, the zone is blue with a dotted pattern. VCGs leading to an inconsistently significant results are red with a crossed pattern. VCGs leading to an inconsistently non-significant results are orange with a checkered pattern. And finally, VCGs leading to an inverse significant result are magenta with a with a diagonally stroked pattern.


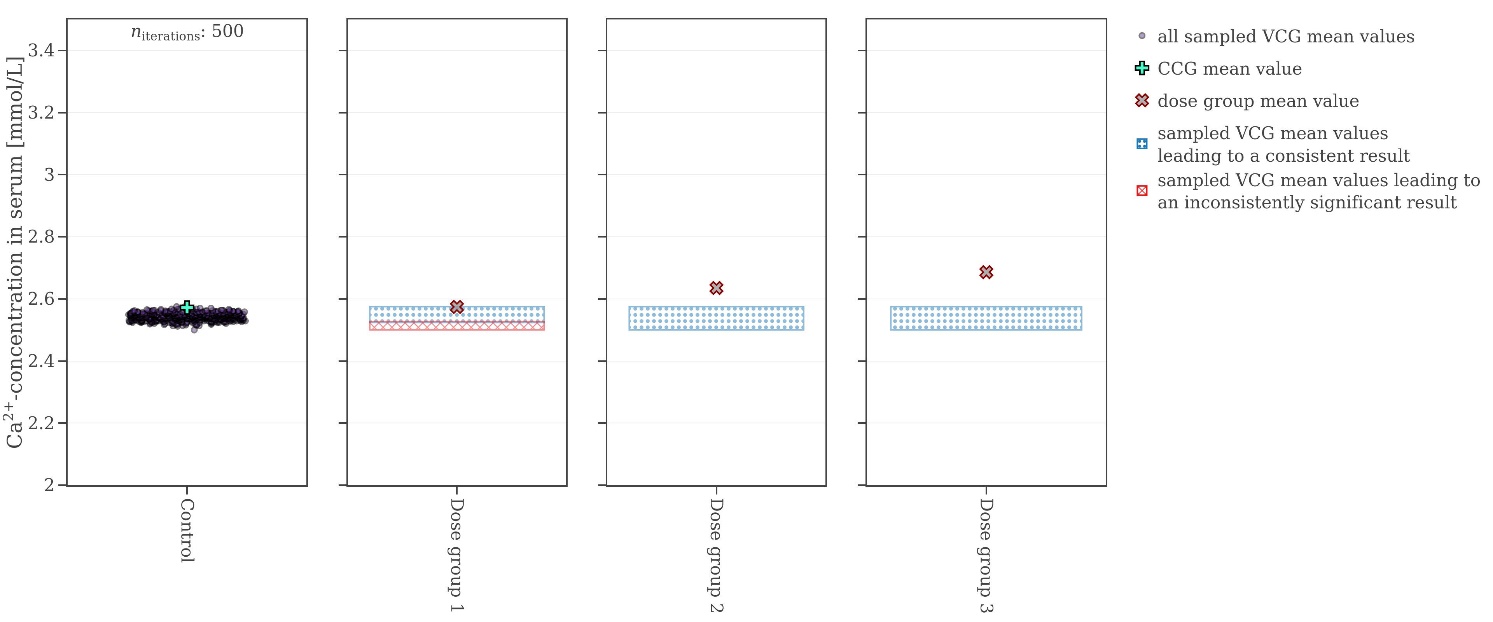


Figure S7: Scenario 1b: confounder not controlled; two sentinel animals kept. Resampling results of the legacy study in male rats in each dose group. Virtual control groups (VCGs) were generated using the calcium sample population of the respective subset The mean value of concurrent control group (CCG) (green cross) and the dose group (grey X) are shown for each dose group. Additionally, all mean values of the sampled VCGs of 500 iterations are shown as a scattered violet cloud in the control-group panel. On the dose groups, the areas for the VCG from each iteration are shown as “zones”. If a VCG led to a result consistent with the one using the CCG, the zone is blue with a dotted pattern. VCGs leading to an inconsistently significant results are red with a crossed pattern. VCGs leading to an inconsistently non-significant results are orange with a checkered pattern. And finally, VCGs leading to an inverse significant result are magenta with a with a diagonally stroked pattern.


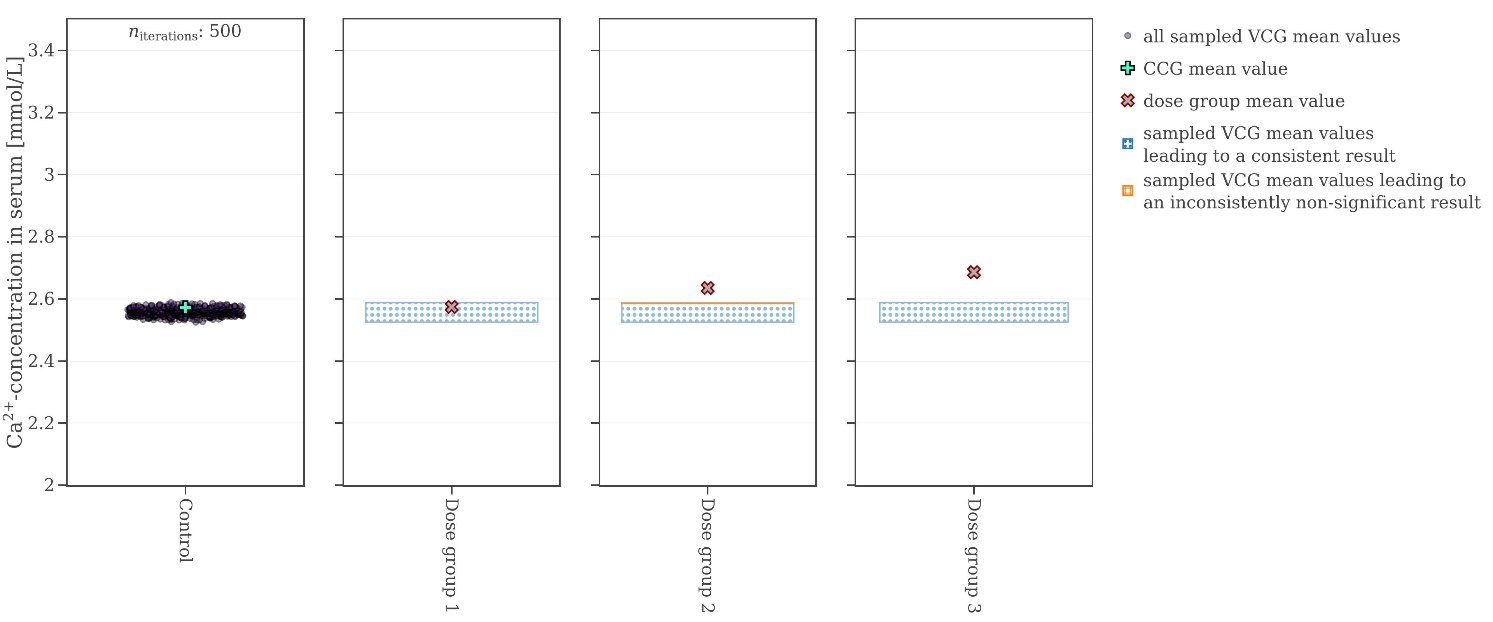


Figure S8: Scenario 1c: confounder not controlled; half of the CCG animals are kept as sentinel animals. Resampling results of the legacy study in male rats in each dose group. Virtual control groups (VCGs) were generated using the calcium sample population of the respective subset The mean value of concurrent control group (CCG) (green cross) and the dose group (grey X) are shown for each dose group. Additionally, all mean values of the sampled VCGs of 500 iterations are shown as a scattered violet cloud in the control-group panel. On the dose groups, the areas for the VCG from each iteration are shown as “zones”. If a VCG led to a result consistent with the one using the CCG, the zone is blue with a dotted pattern. VCGs leading to an inconsistently significant results are red with a crossed pattern. VCGs leading to an inconsistently non-significant results are orange with a checkered pattern. And finally, VCGs leading to an inverse significant result are magenta with a with a diagonally stroked pattern.


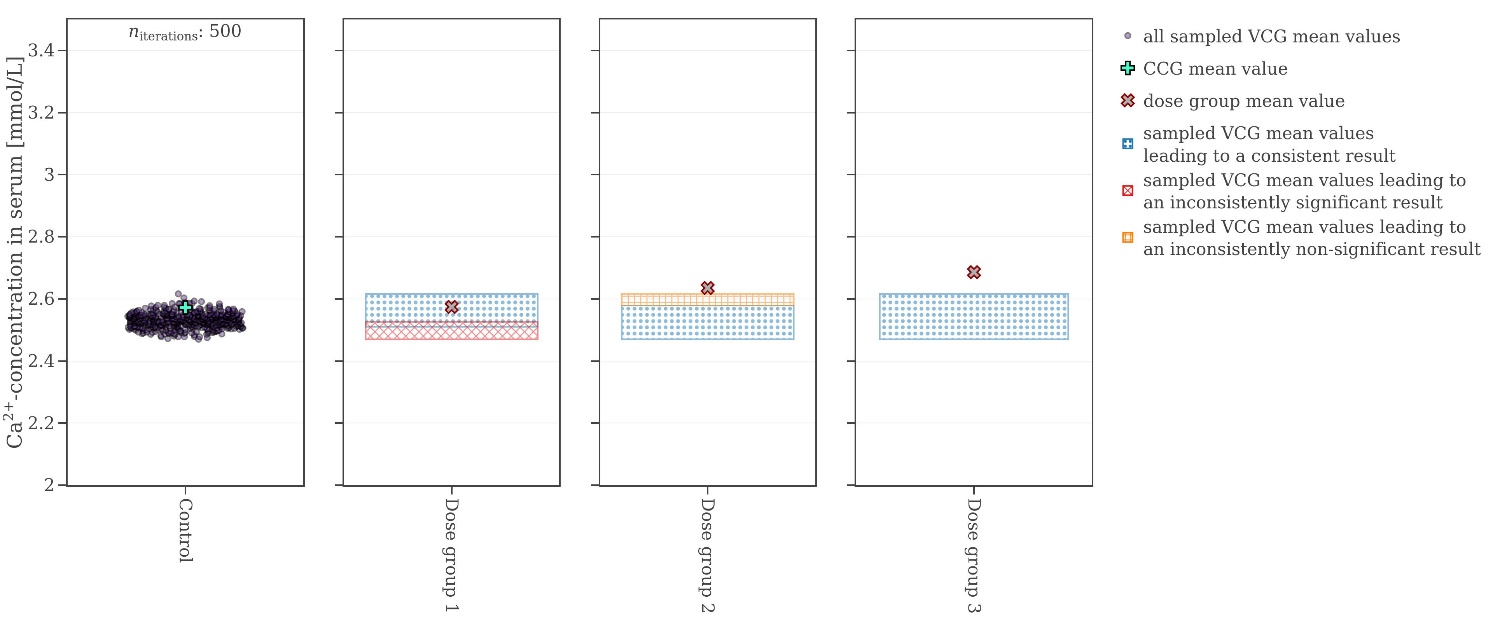


Figure S9: Scenario 2a: confounder controlled; no sentinel animals kept. Resampling results of the legacy study in male rats in each dose group. Virtual control groups (VCGs) were generated using the calcium sample population of the respective subset The mean value of concurrent control group (CCG) (green cross) and the dose group (grey X) are shown for each dose group. Additionally, all mean values of the sampled VCGs of 500 iterations are shown as a scattered violet cloud in the control-group panel. On the dose groups, the areas for the VCG from each iteration are shown as “zones”. If a VCG led to a result consistent with the one using the CCG, the zone is blue with a dotted pattern. VCGs leading to an inconsistently significant results are red with a crossed pattern. VCGs leading to an inconsistently non-significant results are orange with a checkered pattern. And finally, VCGs leading to an inverse significant result are magenta with a with a diagonally stroked pattern.


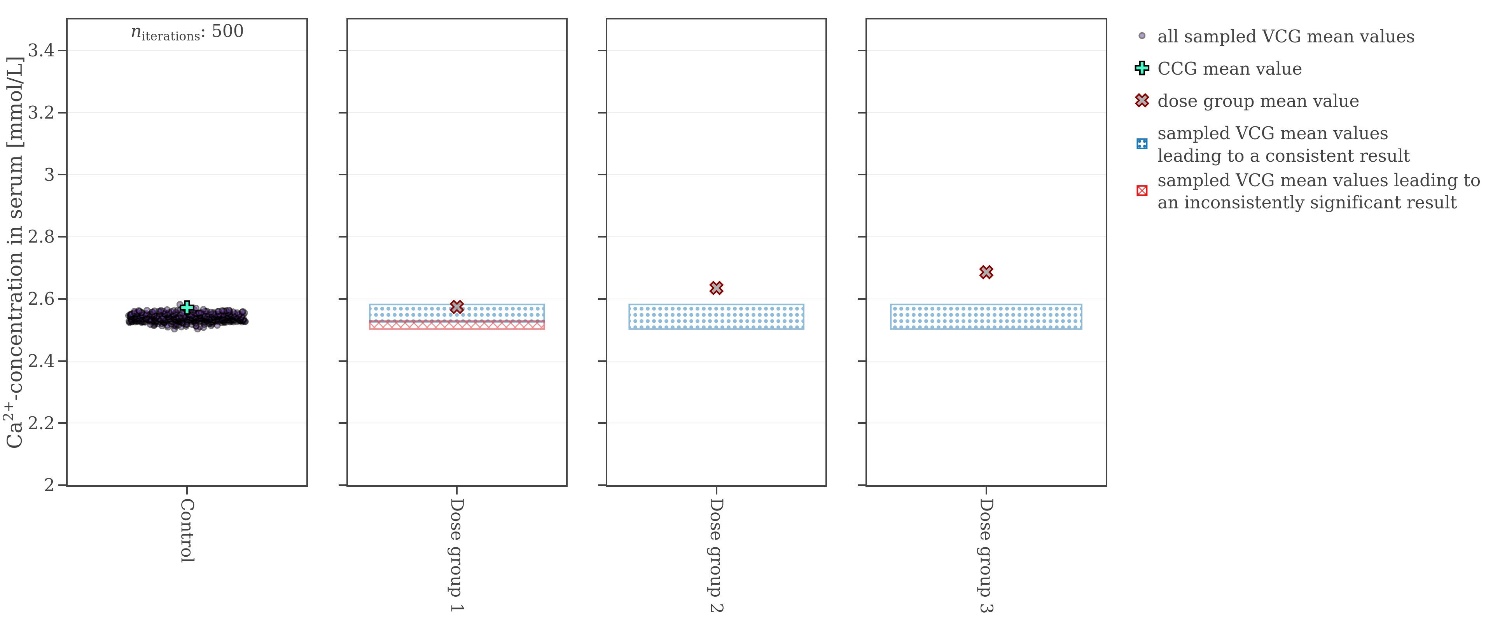


Figure S10: Scenario 2b: confounder controlled; two sentinel animals kept. Resampling results of the legacy study in male rats in each dose group. Virtual control groups (VCGs) were generated using the calcium sample population of the respective subset The mean value of concurrent control group (CCG) (green cross) and the dose group (grey X) are shown for each dose group. Additionally, all mean values of the sampled VCGs of 500 iterations are shown as a scattered violet cloud in the control-group panel. On the dose groups, the areas for the VCG from each iteration are shown as “zones”. If a VCG led to a result consistent with the one using the CCG, the zone is blue with a dotted pattern. VCGs leading to an inconsistently significant results are red with a crossed pattern. VCGs leading to an inconsistently non-significant results are orange with a checkered pattern. And finally, VCGs leading to an inverse significant result are magenta with a with a diagonally stroked pattern.


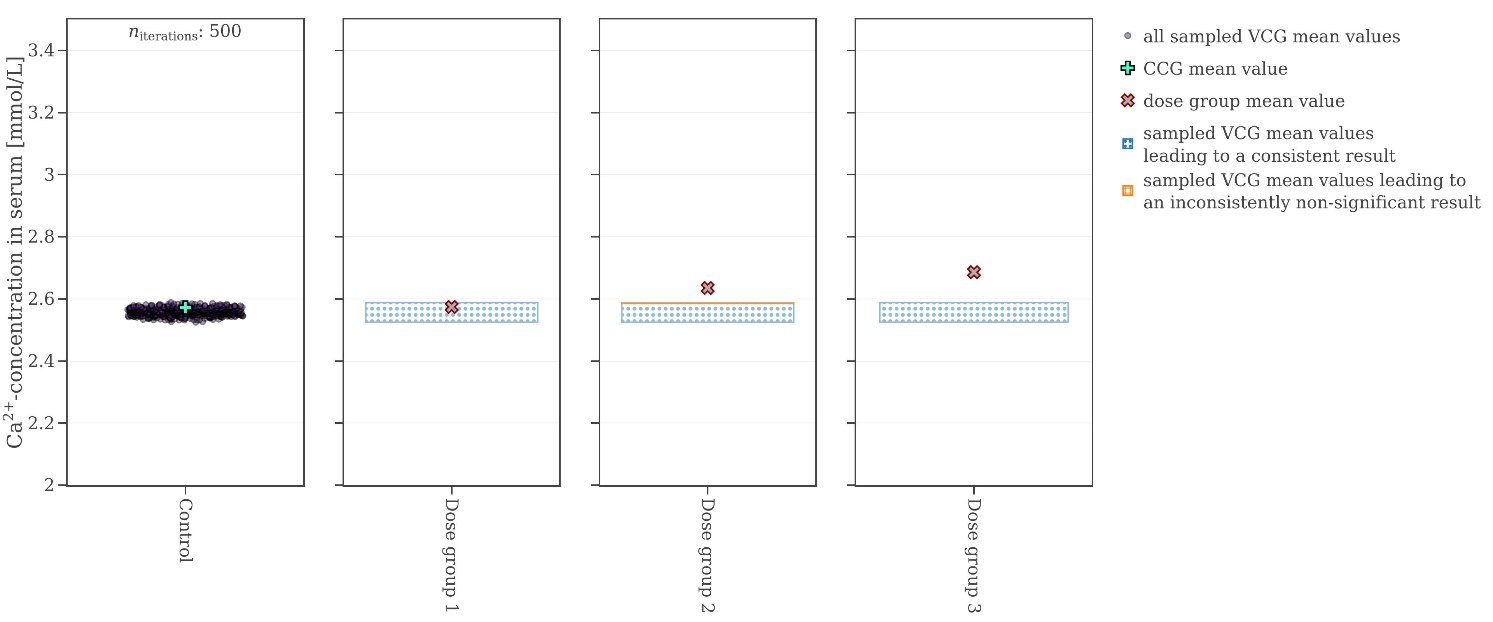


Figure S11: Scenario 2c: confounder controlled; half of the CCG animals are kept as sentinel animals. Resampling results of the legacy study in male rats in each dose group. Virtual control groups (VCGs) were generated using the calcium sample population of the respective subset The mean value of concurrent control group (CCG) (green cross) and the dose group (grey X) are shown for each dose group. Additionally, all mean values of the sampled VCGs of 500 iterations are shown as a scattered violet cloud in the control-group panel. On the dose groups, the areas for the VCG from each iteration are shown as “zones”. If a VCG led to a result consistent with the one using the CCG, the zone is blue with a dotted pattern. VCGs leading to an inconsistently significant results are red with a crossed pattern. VCGs leading to an inconsistently non-significant results are orange with a checkered pattern. And finally, VCGs leading to an inverse significant result are magenta with a with a diagonally stroked pattern.
