# Supplementary material for: Hurdles and signposts on the road to virtual control groups—A case study illustrating the influence of anesthesia protocols on electrolyte levels in rats
Source: Front Pharmacol. 2023 Apr 20;14:1142534. doi: 10.3389/fphar.2023.1142534 (PMC10159271; doi:10.3389/fphar.2023.1142534)
Supplement: Supplementary file 5 [file DataSheet1.docx]

Supplementary Material

# Methods

## The data

All animal studies performed after 2011 were recorded using Pristima (Xybion) Laboratory Information Management System (LIMS). Pristima’s SEND solution, called Savante was utilized for the conversion of the collected raw data into the SEND (Standard for Exchange of Non-clinical Data) data model. Additionally, external SEND studies which were conducted at CROs (Contract Research Organizations) are accessible. Harmonization of the study data was performed according to the SEND controlled terminology for enabling data analysis. The data is being stored in AWS S3 and easily accessible for data scientists at Bayer.

## Used software for data selection, curation, and visualization

Data gathering was performed with a version of the statistical software R, version 3.5.3. The processing, and cleaning of the data was done with R, version 4.0.1, using the tidyverse package (Wickham, 2017) and the data.table package (Dowle and Srinivasan, 2021). Statistical evaluation was performed using the DescTools package (Signorell and al., 2019). Data visualization was performed with, using the plotly package (Sievert, 2018) along with the webshot package for exporting images (Chang, 2019). The used R-code can be downloaded from the GitHub repository https://github.com/bayer-group/VCG-resampling.git.

## Difference between potassium and calcium levels of rats anesthetized with CO_2_/air and CO_2_/O_2_ in the data set

During the procedure of blood drawing in rats, anesthetics are used. One possible anesthetic is CO_2_ which is however not administered purely but as a mixture. In this data set from Bayer, in most cases, CO_2_ was mixed with room air in a 6:4 ratio (60 % CO_2_ and 40 % O_2_). In some studies however, a mixture of 80 % CO_2_ and 20 % O_2_ was used insted. In order to ensure that these additives to the CO_2_ were not influencing the outcome on the electrolyte values, the values were compared to each other. For the potassium values, a histogram (Panel A of Figure S1) and a box plot (Panel B of Figure S1) is illustrated, showing no visible differences between these two subgroups in the potassium values. Same was done for calcium (Figure S2), sodium (Figure S3) and inorganic phosphate (Figure S4).

It was therefore concluded that both sub-sets can be combined into one group as they have no difference in the potassium and calcium values-in relation to the serum values of the rats which were anesthetized with isoflurane.

# Supplementary Figures


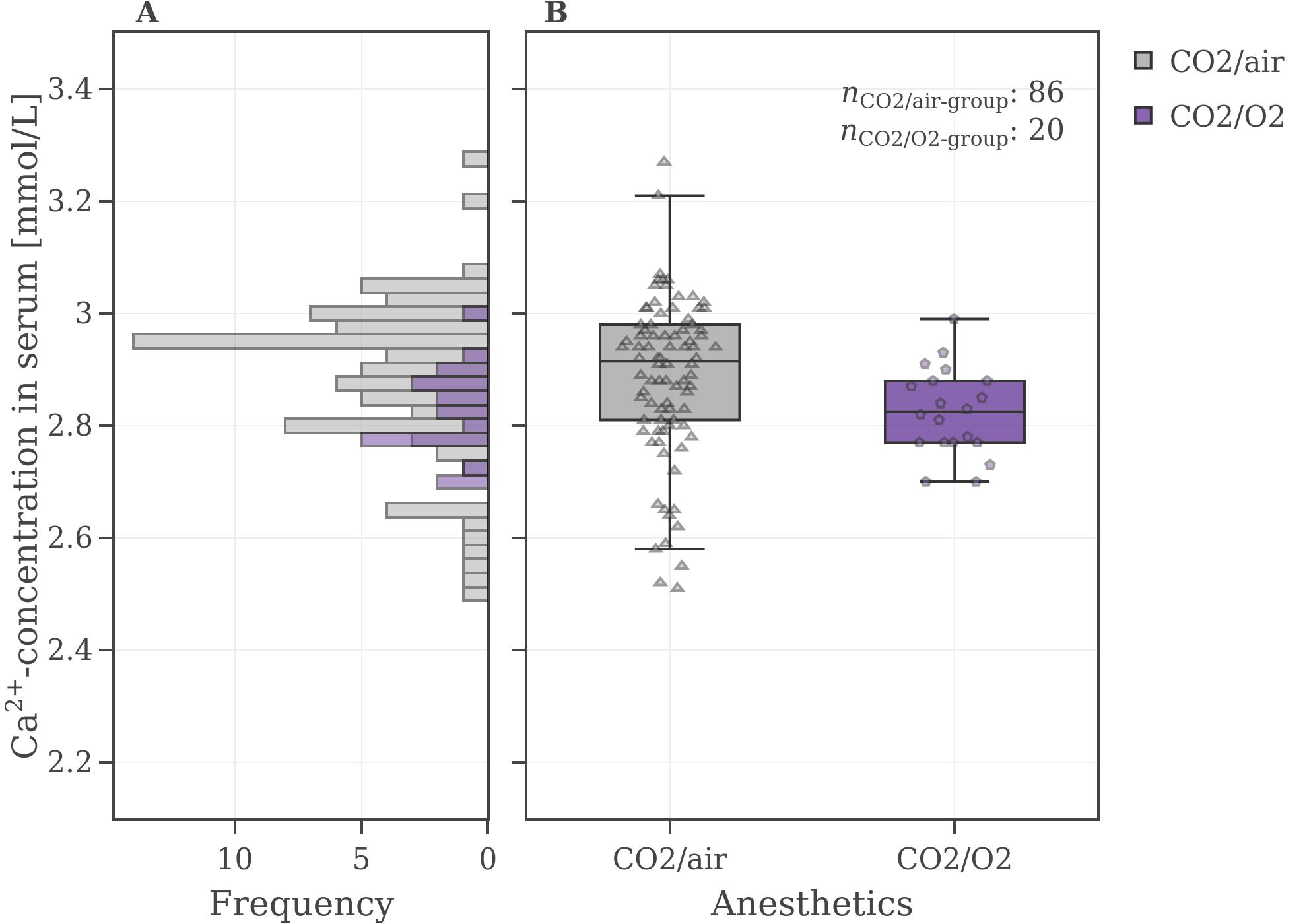


Figure S 1: (A) Calcium value distributions of male Wistar-rats (B) Box plots of these calcium levels with respect to the anesthetic. The CO_2_/air-group is colored grey, and the CO_2_/O_2_-group is colored violet.


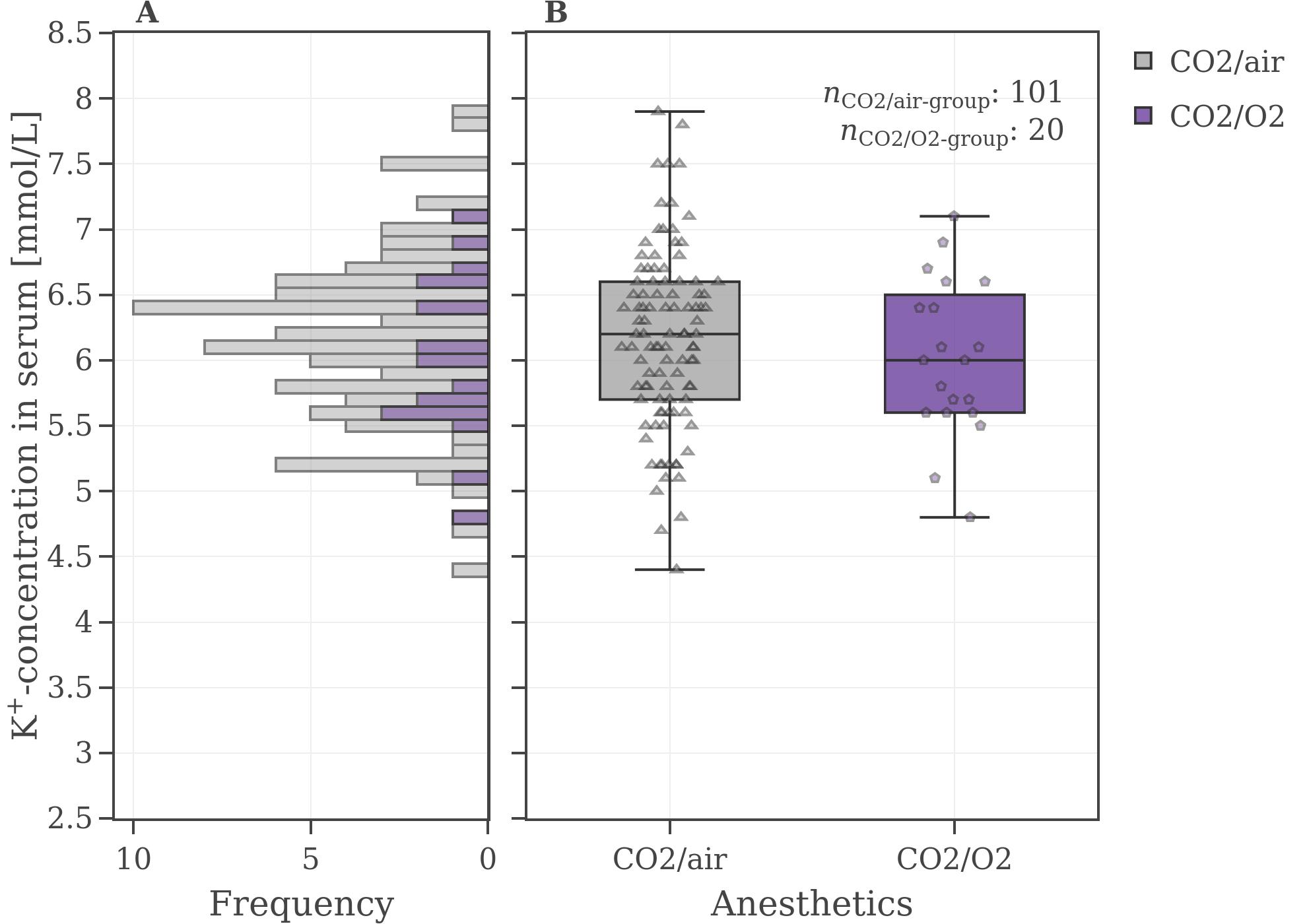


Figure S 2: (A) Potassium value distributions of male Wistar-rats (B) Box plots of these potassium levels with respect to the anesthetic. The CO_2_/air-group is colored grey, and the CO_2_/ O_2_-group is colored violet.


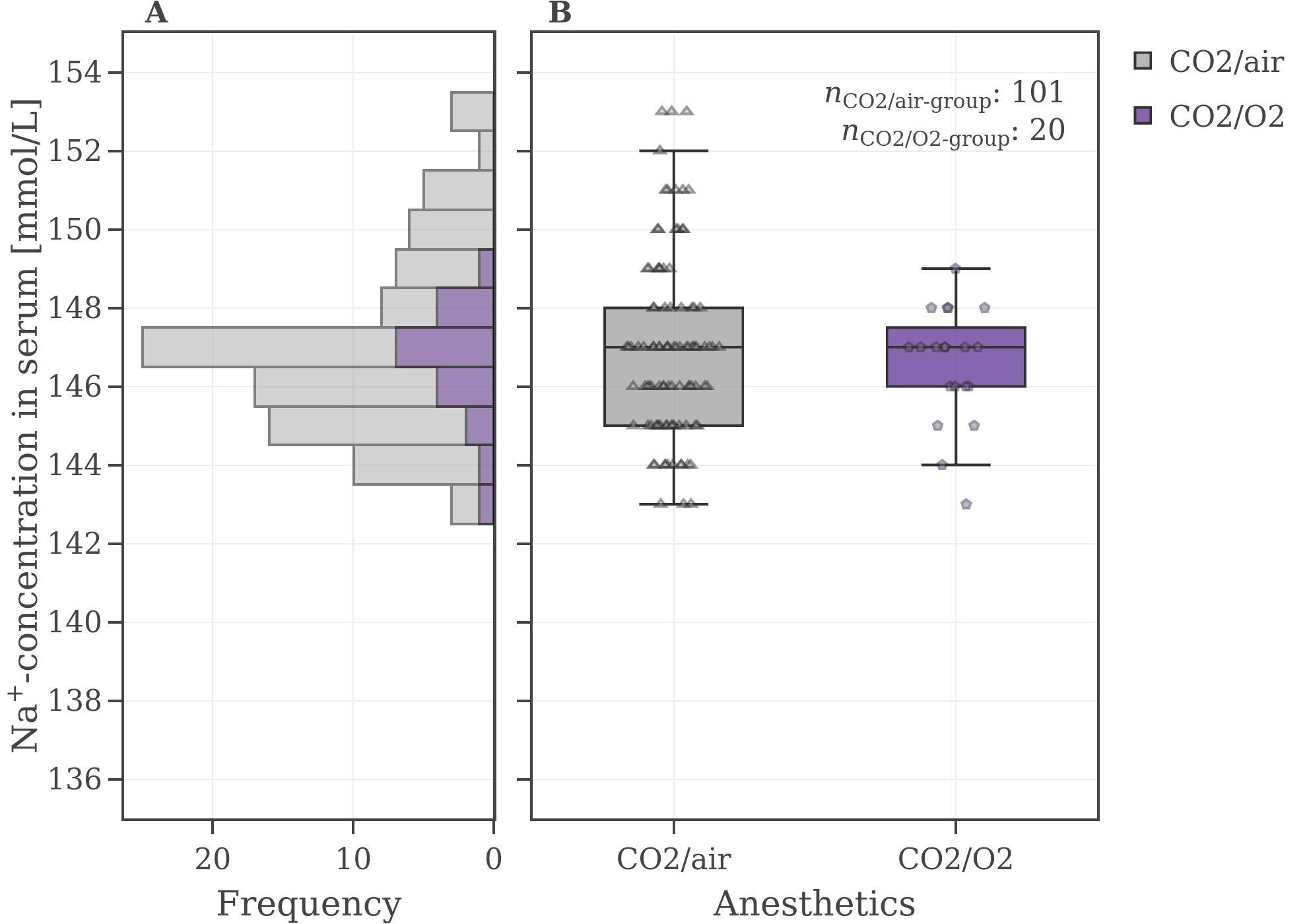


Figure S 3: (A) Sodium value distributions of male Wistar-rats (B) Box plots of these sodium levels with respect to the anesthetic. The CO_2_/air-group is colored grey, and the CO_2_/ O_2_-group is colored violet.


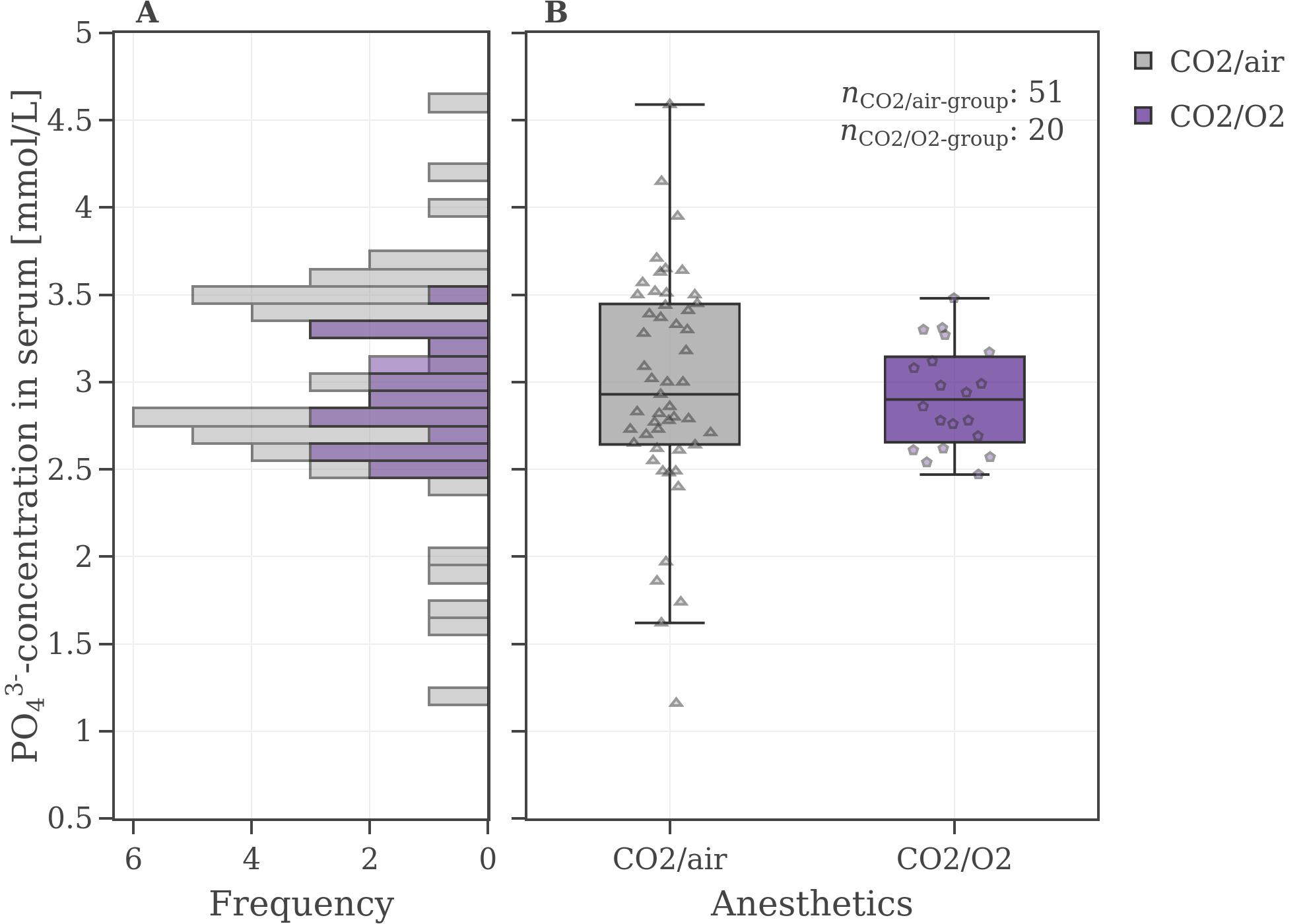


Figure S 4: (A) Phosphate value distributions of male Wistar-rats (B) Box plots of these phosphate levels with respect to the anesthetic. The CO_2_/air-group is colored grey, and the CO_2_/ O_2_-group is colored violet.
